# Assessing and validating the specialized competency framework for pharmacists in sales and marketing (SCF-PSM): a cross-sectional analysis in Lebanon

**DOI:** 10.1186/s40545-023-00638-w

**Published:** 2023-10-27

**Authors:** Joya Namnoum, Aline Hajj, Katia Iskandar, Hala Sacre, Marwan Akel, Rony M. Zeenny, Chadia Haddad, Pascale Salameh

**Affiliations:** 1 Methodology and Statistics in Biomedical Research, Faculty of Medicine, University of Paris-Saclay, Kremlin-Bicêtre, 2 Rue Ambroise Croizat Alfortville, 94140 Créteil, France; 2 INSPECT-LB (Institut National de Santé Publique, d’Épidémiologie Clinique Et de Toxicologie-Liban), Beirut, Lebanon; 3 Faculty of Pharmacy, Université Laval, Québec, Canada; 4 Oncology Division, CHU de Québec- Université Laval Research Center, Québec City, QC Canada; 5 Faculty of Public Health, Lebanese University, Fanar, Lebanon; 6 School of Pharmacy, Lebanese International University, Beirut, Lebanon; 7 Department of Pharmacy, American University of Beirut Medical Center, Beirut, Lebanon; 8 Research Department, Psychiatric Hospital of the Cross, Jal El Dib, Lebanon; 9 School of Medicine, Lebanese American University, Byblos, Lebanon; 10 Faculty of Pharmacy, Lebanese University, Hadath, Lebanon; 11 Department of Primary Care and Population Health, University of Nicosia Medical School, 2417 Nicosia, Cyprus

**Keywords:** Competency framework, Marketing and Sales Pharmacists, Validation, Lebanon

## Abstract

**Objectives:**

Competencies refer to the knowledge, skills, attitudes, and behaviors individuals develop through education, training, and experience. These competencies can be formulated into a framework to support practitioner development for effective and sustained performance. In the absence of a national framework for pharmacy education and practice in Lebanon, the Order of Pharmacists of Lebanon (OPL, the official association of pharmacists in Lebanon) pioneered the development of a pharmacy competency framework in 2017. This study aimed to validate and assess the specialized competency framework for pharmacists in sales and marketing (SCF-PSM) after updating the framework previously published by the OPL. The secondary objective was to assess, in a pilot survey, the personal characteristics associated with these competencies.

**Methods:**

After validating the content of the specialized competency framework, a survey involving Lebanese pharmacists was performed through a 15-min online questionnaire distributed over social media platforms, groups of pharmacists, and individual pharmacists’ contact numbers.

**Key findings:**

Pharmaceutical knowledge, communication, emergency response, and operation management during emergencies were satisfactory (more than 80/100). Other activities during emergencies, such as patient care and population health interventions and evaluation, research, and dissemination of research outcomes, received a moderate score (75–78/100), similar to legal practice (78/100), teamwork (76/100), and management skills (75/100). The lowest reported confidence was related to professional communication skills (other than communication per se), mainly negotiation, data processing skills, information technology, self-management, and ethical practice (< 75/100). This study reported deficiencies between what is acquired during undergraduate, postgraduate, and continuing education on the one hand and the competency framework suggested by the OPL on the other hand, showing a mismatch between the competencies of working pharmacists acquired during education and the market needs.

**Conclusions:**

This study validated a competency framework for pharmacists in sales and marketing and explored the current gaps in self-reported competencies. It also identified areas necessitating reinforcement to optimize professional practice and underscored the need for improvement in undergraduate, postgraduate, and continuing professional education.

**Supplementary Information:**

The online version contains supplementary material available at 10.1186/s40545-023-00638-w.

## Background

A competent practitioner workforce is an essential prerequisite for all healthcare professions. The capacity to improve therapeutic outcomes, patients’ quality of life, scientific advancement, and enhancement of our public health imperatives relies on a foundation of competence. Before overarching capability or competence can be determined, the specific competencies that comprise that capability must be identified [1–3]. In this case, competencies refer to the knowledge, skills, attitudes, and behaviors an individual develops through education, training, and experience [4].

Taken together, these competencies can be formulated into a framework to support practitioner development for effective and sustained performance. Practitioner development frameworks, containing a structured assembly of behavioral competencies, have become increasingly popular in professional education, driven by the need for transparency in training, development, and professional recognition of healthcare professionals. The evidence to support their routine use in professional development is unequivocal [5–7].

Medicine is one of the first health professions to have developed a global competency framework, with the World Federation for Medical Education (WFME)^8^ ensuring that the competencies of physicians are globally applicable, transferable, accessible, and transparent. International standards can be defined for basic medical education while considering the variations of countries due to the differences in teaching, culture, socioeconomic conditions, and health systems, among others [9]. Nonetheless, the scientific basis of medicine remains universal. For the pharmacy profession, the International Pharmaceutical Federation (FIP) Education Initiatives (FIPEd) believes such guidance is also possible for pharmacists and has developed a global competency framework to support the educational development of pharmacy practitioners [10]. The FIP has also recently developed an advanced competency framework [11], although it did not address pharmacy per specialty.

In Lebanon, pharmacists in sales and marketing work in the pharmaceutical industry divisions of sales and marketing, promoting pharmaceuticals in front of medical doctors and other healthcare professionals, and are required to have competencies that differ from those of other pharmacists. These competencies were part of a core competency framework previously suggested by Lebanon’s official Pharmacists’ Association (OPL, Order of Pharmacists of Lebanon)^12^. This framework was inspired by the FIP Global Competency Framework [10] but adapted to meet local needs. It still needs to be officially adopted by the Ministry of Education and Higher Education (MEHE), the Ministry of Public Health (MOPH), and, consequently, universities. It would allow for an evidence-based assessment of competencies and related gaps and help elaborate continuing education and professional development programs. Since this framework was not adopted, it was hypothesized that many gaps still exist between educational programs and practice in the sales and marketing field, particularly at the levels of transferable skills and emergency readiness; these gaps would be detected once graduates started working and would translate into a need for continuous development.

Moreover, with the occurrence of the pandemic and the severe socioeconomic crisis in Lebanon, drug shortages and substandard medications became common, affecting the entire pharmaceutical sector and endangering patient health [13]. This challenging situation led the OPL to suggest a set of competency frameworks for pharmacists of diverse fields of work, i.e., sales and marketing, hospital and clinical pharmacy, industry pharmacy, community pharmacy, and academic preceptorship [14, 15].

The OPL also developed a national pharmaceutical strategy that recommended the validation of specialized competencies per pharmacy specialty [13]. Hence, our team updated the preliminary versions suggested by the OPL and validated and published specialized competency frameworks for different pharmacy sectors [16–20], except for the sales and marketing field. Therefore, this study aimed to validate and assess the specialized competency framework for pharmacists in sales and marketing (SCF-PSM) after updating the framework previously published by the OPL. The secondary objective was to evaluate, in a pilot survey, the personal characteristics associated with these competencies.

## Methods

### Content validation of the framework: expert consensus (phase 1)

An expert panel emanating from the OPL Scientific Committee reviewed the previously suggested framework. The panel included pharmacists from diverse backgrounds, featuring five experts with academic and research experience, including experience in validation and assessment of competency frameworks and two members who brought their expertise in sales, marketing, and management gained from their roles in pharmaceutical companies. The panel reviewed the framework using a Delphi technique, and items were only included if a consensus of 90% was reached among the members. It is paramount to note that the experts deemed it necessary to add a domain related to emergency preparedness and response based on the hardships associated with the COVID-19 pandemic and the socioeconomic crisis in Lebanon [22–25].

The consensual competency framework that was reached included three domains (scales) distributed as follows: pharmaceutical knowledge (6 items without a subscale), professional communication skills (70 items; 11 subscales), and pharmacist preparedness and response (21 items; 4 subscales), with subscales representing competencies while items reflecting their corresponding behaviors. The behavioral statements reported for each competency indicate how pharmacists in sales and marketing would behave in practice. For every behavior, answers were graded on a 5-point Likert scale (very confident, fairly confident, neither/I don’t know, slightly confident, and not confident at all) [Additional file 1].

### Structure validation of the framework: a cross-sectional survey (phase 2)

A cross-sectional pilot survey was conducted in May 2022 to validate the structure of the competency framework and assess its correlates. It involved a probabilistic sample of pharmacists who worked in the sales and marketing field without holding managerial positions, as managers had a specific framework that included competencies [18] that differed from the ones suggested for non-managers. The OPL provided the official list of pharmacists with their mobile numbers. This list included 1336 pharmacists registered as working in the sales and marketing field, of whom 105 pharmacists lived outside Lebanon for more than ten years, and 439 pharmacists had managerial positions and thus were excluded; the data collection targeted the remaining 792 pharmacists. The 15-min self-assessment questionnaire developed on Google Forms was sent to them privately via WhatsApp messages. The final dataset consisted of 231 observations (29.2% acceptance rate).

### Ethical statement

The Lebanese International University Ethics and Research Committee approved this project (Approval number: 2020RC-063-LIUSOP). Before enrolling in the survey, participants were required to read and consent to the study objectives and the average expected time to complete the questionnaire. Participation was voluntary, and pharmacists received no incentive in return for their participation. No follow-up was possible as data were collected anonymously and treated confidentially; participants would only view aggregated results once published and diffused. Results were carefully inspected for duplication, and none was found.

### Sample size calculation

The minimum sample size was calculated using the G-Power software version 3.0.10. The calculated effect size was 0.0526, expecting squared multiple correlations of 0.05 (R^2^ deviation from 0) related to the Omnibus test of multiple regression. The minimum necessary sample was n = 152, considering an alpha error of 5%, a power of 80%, and allowing for 10 predictors to be included in the model.

### Questionnaire and variables

The questionnaire was in English and divided into two parts: the first one included the sociodemographic, education, and professional characteristics, i.e., age, gender, level of education, highest degree related to the main field of work, year of graduation, university of graduation, university of the highest degree, the language of pharmacy education, work location, working days per week, working hours per day, if the pharmacist has another field of work, and percentage of competencies acquired before graduation and after graduation (by continuing education or experience). These data would allow self-assessed competencies to be compared with the variables cited.

The second part consisted of questions to measure the competency framework previously established and included all behaviors reported by the participants.

### Statistical analysis (phase 3)

Data were converted from Excel to RStudio version 4.1.2. First, a single-variable descriptive analysis was performed. Then, the framework was validated by conducting item analysis, correlation matrices (to confirm the structure of the subscales), and dimensionality assessment. Factor loadings were reported after checking data sampling adequacy using the Kaiser–Meyer–Olkin (KMO) test. Reliability was also assessed using the Cronbach alpha; coefficients above 0.6 are considered acceptable for questionnaire surveys [26].

Afterward, means and standard deviations were calculated for every competency (dependent variables) and compared with groups of personal characteristics (independent variables). Means were then compared using the Student’s T-test for two groups and ANOVA for three groups or more in bivariate analysis. Finally, General Linear Models were used to assess correlates of competencies through multivariate analysis, using pharmacists’ personal characteristics as independent variables included in the model through the ENTER method; estimates, standard errors, confidence intervals, and p-values were reported. A P-value less than 0.05 was considered significantly significant.

## Results

### Construct validity assessment

The factor analysis results showed that the pharmaceutical knowledge and professional communication competency scales were unidimensional (results not shown), while the emergency preparedness and response (PP) scale exhibited a bi-dimensional structure (Table 1). All Emergency Preparedness and Response Competencies (PPE) and all Operation Management Competencies (PPO) items (behaviors) showed robust loadings on factor 1, designating it as the "operational aspect" of pharmacists' emergency preparedness. Furthermore, all Patient Care and Population Health Interventions Competencies (PPC) and all Evaluation, Research, and Dissemination for Impact and Outcomes Competencies (PPERD) had their highest loadings on factor 2, which can be labeled as the "clinical and research aspect" of pharmacists' readiness for emergencies. This factor analysis confirms the original two-subscale structure of the PP scale.

**Table 1:** Factor analysis of pharmacists’ preparedness and response in emergency situations

Pharmacists’ preparedness and response in emergency situations scale	Item	Factor 1*	Factor 2**
PPE: emergency preparedness and response	PPE1	**0.501**	0.360
	PPE2	**0.417**	0.303
	PPE3	**0.566**	0.288
	PPE4	**0.597**	0.367
	PPE5	**0.516**	0.428
	PPE6	**0.469**	0.319
	PPE7	**0.463**	0.170
PPO: operation management	PPO1	**0.742**	0.380
	PPO2	**0.775**	0.202
	PPO3	**0.792**	0.272
	PPO4	**0.814**	0.311
	PPO5	**0.864**	0.284
	PPO6	**0.816**	0.281
	PPO7	**0.816**	0.255
	PPO8	**0.766**	0.364
PPC: patient care and population health interventions	PPC1	0.463	**0.544**
	PPC2	0.510	**0.572**
PPERD: evaluation, research, and dissemination for impact and outcomes	PPERD1	0.284	**0.886**
	PPERD2	0.223	**0.902**
	PPERD3	0.276	**0.773**
	PPERD4	0.290	**0.752**

^*^Factor 1: operational aspect

^**^Factor 2: clinical and research aspect. The bold values correspond to the higher loading factor between factor 1 and factor 2

### Reliability analysis

Cronbach alpha coefficients were calculated for the measurement of internal consistency (Table 2); they were higher for Professional Communication Skills (PCS) and PP scales because they were obtained from longer scales (70 items in PCS and 21 items in PP). However, it was smaller for the six-item PK scale since it was the shortest scale. The measurements were reliable (> 0.8) (Table 2).

**Table 2:** Cronbach α for self-reported competency scales of sales and marketing pharmacists

Scale	Sample size	Number of items	Cronbach alpha
Pharmaceutical knowledge	230	6	0.893
Professional communication skills	230	70	0.976
Pharmacist preparedness and response in emergencies	230	21	0.957

### Descriptive analysis

Of the 230 participant pharmacists working in sales and marketing, 71.74% were females. Educational variables showed that 40.87% had a BS in pharmacy as the highest degree related to the main field of work, 14.78% a PharmD, 22.61% a master’s degree, and 2.6% a Ph.D. or equivalent. The highest percentage graduated from Saint-Joseph University of Beirut (USJ; 19.60%), 17.40% from the Lebanese University (UL), which is the only public university in Lebanon, 16.50% from the Lebanese American University (LAU), 16.10% from the Lebanese International University (LIU), 13.00% from the Beirut Arab University (BAU), and 16.6% from other Lebanese and foreign universities.

The majority of participants (66.52%) graduated after 2011, 27.39% between 2000 and 2010, and 6.09% before 2009. Furthermore, 52.61% worked in Beirut, 19.13% in Mount Lebanon, 5.22% in Beqaa, 3.48% in South Lebanon, and 6.96% were not currently working. Regarding their work experience, 68.21% had less than ten years of experience in the sales and marketing field, 26.52% had between 11 and 20 years of experience, and 5.22% had more than 20 years of experience. The mean age of respondents was 34.31 years (SD = 6.51). The majority were English-educated (63%) and not recently graduated (median = 2012); the sample was well distributed among universities and work locations (Additional file 2: Table S1).

**Fig. 1:**
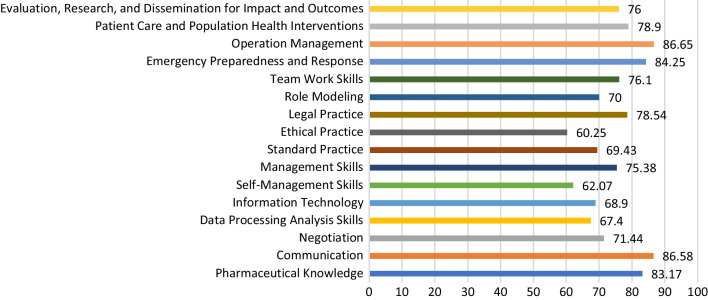
Self-assessment of specialized competencies among sales and marketing pharmacists

Self-declared competencies are described in detail in Additional file 2 (Additional file 2: Tables S2 and S3). Pharmaceutical knowledge, communication, emergency response, and operation management during emergencies were satisfactory (more than 80/100). Other activities during emergencies, such as patient care and population health interventions, and evaluation, research, and dissemination of research outcomes, received a moderate score (75-78/100), similar to legal practice (78/100), teamwork (76/100), and management skills (75/100). The lowest reported confidence was related to professional communication skills (other than communication per se), mainly negotiation, data processing skills, information technology, self-management, and ethical practice (< 75/100) (Fig. 1). Most participants (70%) declared having acquired their competencies through experience (Additional file 2: Table S2).

### Bivariate and multivariate analysis

Bivariate analysis for all competency scales was conducted (Additional file 2: Tables S4, S5, and S6). The detailed multivariable analysis is presented in Additional file 2: Table S7.

Table 3 presents the significant results of the multivariable analysis. Extended experience and acquiring competencies by experience were associated with higher scores on the majority of the scales and subscales while gaining competencies during undergraduate, postgraduate, or continuing education was associated with lower scores.

**Table 3:** Multivariable analysis of sales and marketing self-reported competencies

Variable	Estimate(B)	Standard error	Lower CI	Upper CI	*p*-value
*Pharmaceutical knowledge*					
Highest degree					
BS = 94	Ref				
PharmD/DPharm = 34	− 0.498	0.445	− 1.369	0.381	0.265
Master = 52	− 0.157	0.497	− 1.136	0.816	0.753
Ph.D. or equivalent = 6	0.389	1.201	− 1.972	2.751	0.746
Other degrees (MBA, DBA) = 4	− 5.146	1.461	− 7.995	− 2.262	< 0.001*
*Professional communication skills*					
Communication					
Competencies acquired during undergraduate studies					
[0–25] = 107	Ref				
[26–50] = 79	− 0.336	0.549	− 1.417	0.746	0.541
[51–75] = 29	− 2.566	0.807	− 4.158	− 0.975	0.002*
[76–100] = 15	− 0.205	1.067	− 2.306	1.897	0.848
Competencies acquired during postgraduate studies					
[0–25] = 109	Ref				
[26–50] = 46	0.331	0.636	− 0.921	1.585	0.603
[51–75] = 31	− 1.970	0.840	− 3.625	− 0.315	0.020*
[76–100] = 44	0.024	0.835	− 1.622	1.670	0.977
Competencies acquired during continuing education sessions					
[0–25] = 126	Ref				
[26–50] = 50	0.009	0.612	− 1.197	1.215	0.988
[51–75] = 20	− 2.172	0.935	− 4.015	− 0.328	0.021*
[76–100] = 34	− 0.010	0.949	− 1.881	1.861	0.991
*Negotiation*					
Competencies acquired during postgraduate studies					
[0–25] = 109	Ref				
[26–50] = 46	0.282	0.531	− 0.765	1.328	0.597
[51–75] = 31	− 1.648	0.616	− 2.862	− 0.433	0.008*
[76–100] = 44	0.254	0.543	− 0.817	1.324	0.641
*Data processing analysis*					
Years of experience					
[0–10] = 157	Ref				
[11–20] = 61	1.860	0.762	0.359	3.362	0.015*
[14, 21–29] = 12	2.231	1.721	− 1.161	5.623	0.196
*Information technology*					
Work location					
Beirut = 121	Ref				
Beqaa = 12	0.196	0.541	− 0.871	1.264	0.717
Mount Lebanon = 44	0.891	0.310	0.280	1.503	0.004*
North Lebanon = 29	0.175	0.366	− 0.546	0.897	0.633
South Lebanon = 8	0.240	0.642	− 1.026	1.507	0.708
Currently not working = 16	0.292	0.482	− 0.658	1.242	0.546
*Self-management skills*					
PharmD/DPharm					
No = 138	Ref				
Yes = 92	− 1.096	0.440	− 1.963	− 0.229	0.013*
Competencies acquired during postgraduate studies					
[0–25] = 109	Ref				
[26–50] = 46	0.254	0.571	− 0.872	1.381	0.656
[51–75] = 31	− 1.771	0.665	− 3.082	− 0.460	0.008*
[76–100] = 44	0.034	0.586	− 1.121	1.187	0.955
*Management skills*					
An additional field of work					
Other field pharmacy = 29	Ref				
No other field of work = 192	1.582	0.787	0.032	3.132	0.045*
Other field = 9	2.200	1.483	− 0.722	5.123	0.139
Competencies acquired during postgraduate studies					
[0–25] = 109	Ref				
[26–50] = 46	− 0.070	0.683	− 1.416	1.276	0.918
[51–75] = 31	− 1.922	0.795	− 3.489	− 0.355	0.016*
[76–100] = 44	0.639	0.699	− 0.738	2.017	0.361
*Standard practice*					
Years of experience					
[0–10] = 157	Ref				
[11–20] = 61	0.290	0.535	− 0.766	1.345	0.589
[14, 21–29] = 12	2.825	1.256	0.350	5.300	0.025*
Competencies acquired during postgraduate studies					
[0–25] = 109	Ref				
[26–50] = 46	0.540	0.395	− 0.239	1.320	0.173
[51–75] = 31	− 1.353	0.519	− 2.377	− 0.330	0.010*
[76–100] = 44	− 0.202	0.523	− 1.233	0.828	0.699
Year of graduation					
1989–1999 = 14	Ref				
2000–2010 = 63	2.601	1.139	0.355	4.847	0.023*
2011–2020 = 153	2.235	1.180	− 0.091	4.561	0.059
*Ethical practice*					
Competencies acquired during continuing education sessions					
[0–25] = 126	Ref				
[26–50] = 50	− 0.159	0.260	− 0.671	0.354	0.542
[51–75] = 20	− 0.950	0.373	− 1.686	− 0.214	0.012*
[76–100] = 34	0.096	0.308	− 0.512	0.703	0.756
*Role modeling*					
Years of experience					
[0–10] = 157	Ref				
[11–20] = 61	0.501	0.389	− 0.265	1.268	0.199
[14, 21–29] = 12	2.523	0.902	0.745	4.301	0.006*
Competencies acquired during continuing education sessions					
[0–25] = 126	Ref				
[26–50] = 50	0.008	0.266	− 0.517	0.533	0.977
[51–75] = 20	− 1.092	0.379	− 1.840	− 0.345	0.004*
[76–100] = 34	− 0.023	0.312	− 0.637	0.591	0.940
Year of graduation					
1989–1999 = 14	Ref				
2000–2010 = 63	1.944	0.814	0.339	3.550	0.018*
2011–2020 = 153	2.116	0.845	0.450	3.781	0.013*
Gender					
Female = 165	Ref				
Male = 65	− 0.489	0.233	− 0.950	− 0.029	0.037*
Team working skills					
Working 8.5 h/day	− 0.063	0.022			0.004*
*Pharmacist preparedness and response in emergency situations*					
Emergency preparedness and response					
Competencies acquired during undergraduate studies					
[0–25] = 107	Ref				
[26–50] = 79	− 0.074	0.736	− 1.525	1.377	0.920
[51–75] = 29	0.027	1.074	− 2.090	2.144	0.980
[76–100] = 15	3.505	1.409	0.727	6.284	0.014*
Competencies acquired during postgraduate studies					
[0–25] = 109	Ref				
[26–50] = 46	0.728	0.811	− 0.870	2.326	0.370
[51–75] = 31	2.310	1.097	0.146	4.473	0.036*
[76–100] = 44	0.654	1.078	− 1.472	2.780	0.545
An additional field of work					
Other field of pharmacy = 29	Ref				
No other field of work = 192	− 1.848	0.901	− 3.625	− 0.071	0.042*
Other field	− 3.364	1.702	− 6.719	− 0.009	0.049*
Operation management					
PharmD/DPharm					
No = 138	Ref				
Yes = 92	− 3.850	1.684	− 7.172	− 0.527	0.023*
An additional field of work					
Other than pharmacy = 29	Ref				
No other field of work = 192	− 2.893	1.269	− 5.397	− 0.390	0.024*
Other field	− 6.555	2.332	− 11.156	− 1.953	0.005*
Work location					
Beirut = 121	Ref				
Beqaa = 12	0.782	1.902	− 2.970	4.534	0.681
Mount Lebanon = 44	2.658	1.089	0.510	4.807	0.016*
North Lebanon = 29	0.275	1.331	− 2.350	2.901	0.836
South Lebanon = 8	0.996	2.343	− 3.627	5.620	0.671
Currently not working = 16	3.710	1.732	0.291	7.128	0.033*
*Patient care and population health interventions*					
PharmD/DPharm					
No = 138	Ref				
Yes = 92	− 1.048	0.417	-1.871	− 0.225	0.013*
University					
Foreign University = 18	Ref				
Lebanese American University = 46	− 1.019	0.518	− 2.041	0.003	0.051
Lebanese International University = 46	− 0.977	0.498	− 1.959	0.005	0.051
Saint Joseph University of Beirut = 46	− 1.184	0.524	− 2.218	− 0.150	0.025
Beirut Arab University = 39	− 1.289	0.497	− 2.269	− 0.309	0.010*
Lebanese University = 32	− 0.571	0.542	− 1.640	0.497	0.293
Competencies acquired during undergraduate studies					
[0–25] = 107	Ref				
[26–50] = 79	0.266	0.290	− 0.306	0.838	0.361
[51–75] = 29	0.240	0.406	− 0.561	1.042	0.555
[76–100] = 15	1.186	0.537	0.127	2.245	0.028*
*Evaluation, research, and dissemination for impact and outcomes*					
University					
Foreign University = 18	Ref				
Lebanese American University = 46	− 1.263	1.021	− 3.277	0.750	0.217
Lebanese International University = 46	− 2.355	0.988	− 4.304	− 0.407	0.018*
Saint Joseph University of Beirut = 46	− 1.441	1.076	− 3.562	0.680	0.182
Beirut Arab University = 39	− 1.795	1.010	− 3.787	0.196	0.077
Lebanese University = 32	− 1.109	1.117	− 3.311	1.094	0.322
PharmD/DPharm					
No = 138	Ref				
Yes = 92	− 1.835	0.862	− 3.534	− 0.135	0.034*
Competencies acquired during undergraduate studies					
[0–25] = 107	Ref				
[26–50] = 79	0.822	0.597	− 0.355	1.998	0.170
[51–75] = 29	0.714	0.839	− 0.940	2.368	0.396
[76–100] = 15	2.417	1.112	0.224	4.609	0.031*
Competencies acquired during postgraduate studies					
[0–25] = 109	Ref				
[26–50] = 46	− 1.552	0.654	− 2.841	− 0.263	0.018*
[51–75] = 31	0.099	0.866	− 1.609	1.808	0.909
[76–100] = 44	0.894	0.872	− 0.826	2.614	0.307
Competencies acquired during continuing education sessions					
[0–25] = 126	Ref				
[26–50] = 50	1.275	0.654	0.084	2.465	0.018*
[51–75] = 20	− 0.850	0.866	− 2.739	1.038	0.909
[76–100] = 34	− 0.341	0.872	− 2.232	1.550	0.307

*BS* Bachelor of Science*; CE* continuing education*; CI* confidence interval*; MBA* Master of Business Administration. Values marked in bold are significant *p* < 0.05

A significantly lower pharmaceutical knowledge was found among pharmacists who reported MBA as the highest degree related to their main field of work and those who considered that most (51% to 75%) of their communication competencies were acquired during undergraduate studies, followed by those who gained them during continuing education and postgraduate studies. A significantly lower negotiation competency was found among those who indicated that the majority of their negotiation competencies were acquired during postgraduate studies.

A significantly higher data processing analysis competency was found with increased years of experience, while a significantly higher information technology competency was found in those who worked in Mount Lebanon. A significantly lower self-management skills score was found in PharmD pharmacists and those who considered that most of their self-management skills were acquired during postgraduate studies. A significantly lower management skills competency was found among those considering that most management skills were acquired during postgraduate studies, while significantly higher management skills were found among pharmacists who do not have another field of work.

As for practice, pharmacists who considered that most of their standard practice competencies were acquired during postgraduate studies had a significantly lower standard practice competency, while those who had 21 to 30 years of experience and those who graduated between 2000 and 2010 demonstrated higher standard practice competency. A significantly lower ethical practice competency was found among participants who reported that 51% to 75% of their ethical practice competencies were acquired by continuing education. No significant association was found between legal practice competency and pharmacists’ sociodemographic characteristics.

Regarding communication skills, lower role modeling competency was found among male pharmacists, those who graduated between 2011 and 2020, and those who reported that most of their role modeling competencies were acquired by continuing education. Furthermore, a significantly higher role modeling competency was found among pharmacists who had 21 to 30 years of experience and those who graduated between 2000 and 2010. A significantly higher teamwork skills competency was found in those who worked an average of 8.5 h per day.

A significantly lower emergency and preparedness response competency was found among pharmacists who had another field of work, while a significantly higher emergency and preparedness response competency was found in those who had no other field of work, who considered that more than 75% of their competencies were acquired during undergraduate studies and those who reported that 51% to 75% of their competencies were acquired during postgraduate studies. A significantly lower operation management competency was found in pharmacists with a PharmD degree and those who had another field of work, while higher operation management competency was found among those who had no other field of work, those who worked in Mount Lebanon, and those who were not currently working. A significantly lower patient care and population health interventions competency was found in pharmacists who held a PharmD and those who graduated from BAU and USJ, while a significantly higher patient care and population health interventions competency was found among pharmacists who considered that more than 75% of these competencies were acquired during undergraduate studies. Finally, a significantly lower evaluation, research, and dissemination for impact and outcomes competency was found among pharmacists who had PharmD and those who graduated from LIU, while this competency was significantly higher in pharmacists who considered that more than 76% of these competencies were acquired during undergraduate studies (Table 3).

## Discussion

This study pioneered the content and structure validation of a competency framework for pharmacists in sales and marketing. The Pharmaceutical Knowledge and Professional Communication competency domains were unidimensional, with their items all loading on their respective unique related factor, while the Emergency Preparedness and Response domain was bi-dimensional. The domains were structurally related and reliable. The satisfactory properties of the framework would allow its use in future studies.

The results indicate that pharmacists demonstrated strong proficiency in Pharmaceutical Knowledge, Communication, Emergency Preparedness and Response, and Operation Management competencies, with scores exceeding 80%. However, areas for improvement emerged, with levels below 80% in the following competencies: Negotiation, Data Processing Analysis Skills, Information Technology, Self-Management Skills, Management Skills, Standard Practice, Ethical and Legal Practices, Role Modeling, Team Work Skills, Patient Care and Population Health Interventions, and Evaluation, Research, and Dissemination for Impact and Outcomes. Overall, extended experience and the acquisition of competencies during professional experience were associated with higher scores on most scales and subscales, while gaining competencies during undergraduate, postgraduate, or continuing education was associated with lower scores. Thus, the mismatch between the competencies acquired through education and job needs confirms our hypothesis.

It is worth noting that our team has previously explored the self-declared competencies of pharmacists in Lebanon in different contexts [16–20]. Indeed, in 2020, a study of core competencies showed that, upon their graduation, all pharmacists had the lowest scores in the fundamental knowledge and medicine supply domains and the highest scores in professional practice, personal skills, and safe and rational use of medicines, corresponding to moderate knowledge overall [27]. Comparing these results with our findings is difficult since the previous study was conducted among all newly graduated pharmacists, who may have different perceptions of their competencies, while the current work targeted Lebanese pharmacists in the sales and marketing field that has been highly affected by the dual economic and COVID-19 crises. Nevertheless, all the studies conducted among pharmacists from other professional backgrounds unveiled a clear gap in emergency readiness, in addition to public health interventions, transferable skills, and practice-related competencies [16–19]; this universal finding confirms the results of our work.

Overall, the majority of competencies related to communication skills seemed to be acquired through experience, aligning with the results of a study conducted at the University of Lagos in 2009, where the majority of graduates would improve their communication skills over time after graduation [28]. However, pharmacists who declared having acquired their competencies during undergraduate/postgraduate studies had lower confidence levels. This finding is of utmost importance since it demonstrates divergences between what is gained during undergraduate, postgraduate, and continuing education on the one hand and the suggested sales and marketing competency framework on the other hand, further highlighting the mismatch between learning outcomes and the needed competencies in the work field. Moreover, pharmacists who gained their competencies through continuing education sessions had gaps at several levels, showing the insufficiently/inappropriately designed efforts of professional development.

Our study showed insufficient self-management skills, management skills, and standard practice competencies among pharmacists who had acquired most of their competencies during postgraduate studies. Similarly, a study in Kuwait showed that graduates are ready to implement the various aspects of pharmaceutical care, with the least preparedness in the administrative/management aspects [29]. Another study from Canada found that the lower importance rankings were relatively equally distributed across the manager, advocate, and scholar domains [30]. These findings warrant the integration of communication and managerial skill courses into undergraduate and postgraduate programs.

Furthermore, our results showed low competency scores in patient care and population health interventions, evaluation, research, and dissemination for impact and outcomes among pharmacists who reported that most of these competencies were acquired during their undergraduate studies. This finding aligns with the results of a study evaluating Emergency Preparedness for the COVID-19 pandemic conducted in Malaysia in 2022; this study revealed that the years of professional experience of the respondents were positively correlated with their perceived response to the pandemic [31]. Thus, universities and pharmaceutical companies that recruit pharmacists should have appropriate continuing education programs to equip pharmacists with adequate knowledge and preparedness competencies in emergencies; moreover, universities are encouraged to include these competencies in their undergraduate and postgraduate curricula, as emphasized by the COVID-19 pandemic.

Based on the present findings, the suggested and validated framework is expected to bridge the gap between academia and the labor market once officially adopted and implemented by the MEHE, the MPH, and, consequently, universities, where changes are recommended at the undergraduate and postgraduate levels [27]. At the undergraduate level, improving knowledge and management skills are necessary without decreasing other aspects. Otherwise, at the graduate level, encouraging graduates to pursue specialized education is essential, particularly in Lebanon, where there is an oversupply of non-specialized pharmacists and a concurrent lack of specialized pharmacists [32, 33]. Moreover, continuing education adjustments need to be organized by the OPL and other pharmaceutical institutions that offer such programs^34^, switching towards more tailored professional development activities.

## Limitations and strengths of the study

Although based on a probabilistic method, our sampling was done through an online survey, which might lead to a selection bias that cannot be ruled out, given the particularly lengthy questionnaire that would preclude some busy pharmacists from filling it out. Thus, the results should be interpreted with caution. Moreover, our results can be limited by information bias since the data were self-reported, and pharmacists were explicitly asked to answer about their competencies. Respondents could have also misunderstood the concept of self-assessment or may have overestimated or underestimated their competencies, not to forget the potential burnout that pharmacists could have faced during the economic crisis that has widely affected the pharmaceutical sector; thus, recall bias is also possible. Residual confounding and confounding bias could not be excluded, although the multivariate analysis was performed to account for potential confounders. Multivariate analysis also showed that the normality of some models was sometimes of borderline shape, which decreases their robustness. Lastly, the low response rate of 29% can also limit this study, as pharmacists who participated might be systemically different from those who did not participate. Consequently, it is recommended that further studies be carried out on a larger scale, considering the abovementioned limitations.

Nevertheless, in our study, the majority of respondents were females, with a ratio of 71.74/28.26, representing the actual gender distribution of pharmacists in Lebanon, according to the OPL figures (female 70/30), and this matching in the gender profile makes the study more robust. In addition, implementing modalities to measure and support the development of competencies is a relatively new concept but essential for program enhancement and, subsequently, better pharmacy practice in Lebanon. The value of this competency framework lies in its ability to bridge international pharmacy standards and professional organizations (such as the OPL) and its adaptability to local needs to advance pharmacy practice in Lebanon and bring forward its evolving and dynamic role in the sales and marketing field. Adopting and implementing this framework would guide universities and pharmaceutical companies through the identified gaps to improve professional performance and develop expertise.

## Conclusion

This study validated a competency framework for pharmacists in sales and marketing and explored the current gaps in self-reported competencies. It also identified areas necessitating reinforcement to optimize professional practice, underscoring a mismatch between education and practice-needed skills in professional communication and emergency preparedness. Hence, it becomes compulsory to redesign undergraduate, postgraduate, and continuing education programs to align with these identified needs.

### Supplementary Information

**Additional file 1. **Advanced Competencies for Sales and Marketing Pharmacists questionnaire.

**Additional file 2: Table S1.** Descriptive statistics of sociodemographic variables**. Table S2.** Description of competencies in the sample**. Table S3.** Description of the specialized competencies of sales and marketing pharmacists. **Table S4. **Bivariate Analysis *– Correlates of Pharmaceutical Knowledge***. Table S5. **Bivariate Analysis *– Correlates of Professional Communication Skills***. Table S6.** Bivariate Analysis *– Correlates of Pharmacists’ Preparedness and Response in Emergency Situations***. Table S7.** Multivariable analysis of sales and marketing competencies.

## Data Availability

Data are available upon request.

## Abbreviations

BAU: Beirut Arab University
CI: Confidence interval
FIP: International Pharmaceutical Federation
Lang: Language of education
MBA: Master of Business Administration
MEHE: Ministry of Education and Higher Education
MPH: Ministry of Public Health
OPL: Order of Pharmacists in Lebanon
PCS: Professional communication skills
PK: Pharmaceutical knowledge
PCSC: Communication
PCSD: Data processing analysis
PCSE: Ethical practice
PCSL: Legal practice
PCSMS: Management skills
PCSN: Negotiation
PCSR: Role modeling
PCSSMS: Self-management skills
PCST: Team working skills
PCSP: Standard practice
PPC: Patient care and population health interventions
PPE: Emergency preparedness and response
PPERD: Evaluation, research, and dissemination for impact and outcomes
PPO: Operation management
PP: Pharmacist preparedness and response in emergency situations
USJ: Saint-Joseph University of Beirut
