# Correction: Assessing and validating the specialized competency framework for pharmacists in sales and marketing (SCF-PSM): a cross-sectional analysis in Lebanon

**DOI:** 10.1186/s40545-023-00647-9

**Published:** 2023-11-07

**Authors:** Joya Namnoum, Aline Hajj, Katia Iskandar, Hala Sacre, Marwan Akel, Rony M. Zeenny, Chadia Haddad, Pascale Salameh

**Affiliations:** 1 Methodology and Statistics in Biomedical Research, Faculty of Medicine, University of Paris-Saclay, Kremlin-Bicêtre, 2 Rue Ambroise Croizat Alfortville, 94140 Créteil, France; 2 INSPECT-LB (Institut National de Santé Publique, d’Épidémiologie Clinique Et de Toxicologie-Liban), Beirut, Lebanon; 3 Faculty of Pharmacy, Université Laval, Québec, Canada; 4 Oncology Division, CHU de Québec-Université Laval Research Center, Québec City, QC Canada; 5 Faculty of Public Health, Lebanese University, Fanar, Lebanon; 6 School of Pharmacy, Lebanese International University, Beirut, Lebanon; 7 Department of Pharmacy, American University of Beirut Medical Center, Beirut, Lebanon; 8 Research Department, Psychiatric Hospital of the Cross, Jal El Dib, Lebanon; 9 School of Medicine, Lebanese American University, Byblos, Lebanon; 10 Faculty of Pharmacy, Lebanese University, Hadath, Lebanon; 11 Department of Primary Care and Population Health, University of Nicosia Medical School, 2417 Nicosia, Cyprus

**Correction** **:** **Journal of Pharmaceutical Policy and Practice (2023) 16:128** **https://doi.org/10.1186/s40545-023-00638-w**

Following publication of the original article [1], it was reported that the given and family names of each author were transposed. The original author list incorrectly listed the authors as follows: Namnoum Joya, Hajj Aline, Iskandar Katia, Sacre Hala, Akel Marwan, Zeenny M. Rony, Haddad Chadia and Salameh Pascale.

The correct authorship is given in this Correction article and the original article has been updated.
