# Supplementary material for: Assessing and validating the specialized competency framework for pharmacists in sales and marketing (SCF-PSM): a cross-sectional analysis in Lebanon
Source: J Pharm Policy Pract. 2023 Oct 27;16:128. doi: 10.1186/s40545-023-00638-w (PMC10605801; doi:10.1186/s40545-023-00638-w)
Supplement: Supplementary file 2 — Additional file 2: Table S1. Descriptive statistics of sociodemographic variables. Table S2. Description of competencies in the sample. Table S3. Description of the specialized competencies of sales and marketing pharmacists. Table S4. Bivariate Analysis – Correlates of Pharmaceutical Knowledge. Table S5. Bivariate Analysis – Correlates of Professional Communication Skills. Table S6. Bivariate Analysis – Correlates of Pharmacists’ Preparedness and Response in Emergency Situations. Table S7. Multivariable analysis of sales and marketing competencies. [file 40545_2023_638_MOESM2_ESM.docx]

**APPENDIX**

**Table S1:** Descriptive statistics of sociodemographic variables

| Variable | Subgroups | | N | n (%) | Median | Min-Max | Mean-SD |
| --- | --- | --- | --- | --- | --- | --- | --- |
| **Demographic data** | | | | | | | |
| **Age** | - | | 230 | - | 33 | 24-67 | 34.31-6.51 |
| **Gender** | Female | | 230 | 71.74% (165) | - | - | - |
|  | Male | |  | 28.26% (65) |  |  |  |
| **Educational Data** | | | | | | | |
| **Level of Education** | | | | | | | |
| **BS** | Yes | | 230 | 85.22% (196) | - | - | - |
|  | No | |  | 14.78% (34) |  |  |  |
| **PharmD/**  **DPharm** | Yes | | 230 | 40.00% (92) | - | - | - |
|  | No | |  | 60.00% (138) |  |  |  |
| **Master** | Yes | | 230 | 31.30% (72) | - | - | - |
|  | No | |  | 68.70% (158) |  |  |  |
| **PhD** | Yes | | 230 | 3.48% (8) | - | - | - |
|  | No | |  | 96.52% (222) |  |  |  |
| **Highest degree related to the main field of work** | | | | | | | |
| **HighDeg** | BS Pharmacy | | 230 | 40.87% (94) | - | - | - |
|  | PharmD/DPharm | |  | 14.78% (34) |  |  |  |
|  | Master’s Degree | |  | 22.61% (52) |  |  |  |
|  | PhD or equivalent | |  | 2.60% (6) |  |  |  |
|  | Others | |  | 1.74% (4) |  |  |  |
| **Year of Graduation** | | | | | | | |
| **YearGrad** | 1989-1999 | | 230 | 6.09% (14) | 2012 | 1989-2022 | 2011-6.05 |
|  | 2000-2010 | |  | 27.39% (63) |  |  |  |
|  | 2011-2020 | |  | 66.52% (153) |  |  |  |
| **University of graduation as a pharmacist** | | | | | | | |
| **UniPh** | Lebanese American University | | 230 | 20.00% (46) | - | - | - |
|  | Lebanese International University | |  | 20.00% (46) |  |  |  |
|  | Saint Joseph University of Beirut | |  | 20.00% (46) |  |  |  |
|  | Beirut Arab University | |  | 17.00% (39) |  |  |  |
|  | Lebanese University | |  | 13.90% (32) |  |  |  |
|  | Foreign | |  | 7.80% (18) |  |  |  |
|  | NA | |  | 1.30% (3) |  |  |  |
| **University of the highest degree** | | | | | | | |
| **UniHighDeg** | Lebanese American University | | 230 | 16.50% (38) | - | - | - |
|  | Lebanese International University | |  | 16.10% (37) |  |  |  |
|  | Saint Joseph University of Beirut | |  | 19.60% (45) |  |  |  |
|  | Beirut Arab University | |  | 13.00% (30) |  |  |  |
|  | Lebanese University | |  | 17.40% (40) |  |  |  |
|  | OtherLeb | |  | 8.30% (19) |  |  |  |
|  | OtherForeign | |  | 8.30% (19) |  |  |  |
|  | NA | |  | 0.90% (2) |  |  |  |
| **Language of pharmacy education** | | | | | | | |
| **Lang** | English | | 230 | 63.50% (146) | - | - | - |
|  | French | |  | 33.90% (78) |  |  |  |
|  | Other | |  | 2.60% (6) |  |  |  |
| **Work Data** | | | | | | | |
| **Work Location** | | | | | | | |
| **WLoc** | | Beirut | 230 | 52.61% (121) | - | - | - |
|  |  | Beqaa |  | 5.22% (12) |  |  |  |
|  |  | Mount Lebanon |  | 19.13% (44) |  |  |  |
|  |  | North Lebanon |  | 12.61% (29) |  |  |  |
|  |  | South Lebanon |  | 3.48% (8) |  |  |  |
|  |  | Currently not working |  | 6.96% (16) |  |  |  |
| **Number of working days per week** | | | | | | | |
| **Wdays/wk** | | 0 | 230 | 1.22% (5) | 5 | 0-6 | 4.9-0.8 |
|  |  | 2 |  | 0.44% (1) |  |  |  |
|  |  | 3 |  | 0.88% (2) |  |  |  |
|  |  | 5 |  | 93.04% (214) |  |  |  |
|  |  | 6 |  | 3.48% (8) |  |  |  |
| **Number of working hours per day** | | | | | | | |
| **Whrs/dy** | | 0 | 230 | 2.17% (5) | 8 | 0-24 | 8.21 2.53 |
|  |  | 4 |  | 0.44% (1) |  |  |  |
|  |  | 6 |  | 2.17% (5) |  |  |  |
|  |  | 7 |  | 7.83% (18) |  |  |  |
|  |  | 8 |  | 64.34% (148) |  |  |  |
|  |  | 8.5 |  | 0.88% (2) |  |  |  |
|  |  | 9 |  | 17.39% (40) |  |  |  |
|  |  | 10 |  | 2.60% (6) |  |  |  |
|  |  | 12 |  | 0.44% (1) |  |  |  |
|  |  | 24 |  | 1.74% (4) |  |  |  |
| **Number of years of practicing as a sales/marketing pharmacist** | | | | | | | |
| **YrsExp** | | 0-10 | 230 | 68.26% (157) | 8 | 0-30 | 8.21-2.53 |
|  |  | 11-20 |  | 26.52% (61) |  |  |  |
|  |  | 21-30 |  | 5.22% (12) |  |  |  |
| **Availability of another field of work** | | | | | | | |
| **Work2** | | NoOtherFieldofWrk | 230 | 83.48% (192) |  |  |  |
|  |  | OtherFieldPh |  | 12.61% (29) |  |  |  |
|  |  | OtherField |  | 3.91% (9) |  |  |  |

**Table S2:** Description of competencies in the sample

| **Competencies for Sales/Marketing Pharmacists** | | | |
| --- | --- | --- | --- |
| Variable | Subgroups | N | n (%) |
| **PK = Pharmaceutical Knowledge** | | | |
| **PK1 =** Have thorough knowledge of the different categories of pharmaceuticals, and the therapeutic value of each drug category | Very confident | 230 | 64.78% (149) |
|  | Fairly confident |  | 32.6% (75) |
|  | Neither/I don’t know |  | 0.44% (1) |
|  | Slightly confident |  | 1.74% (4) |
|  | Not confident at all |  | 0.44% (1) |
| **PK2 =** Provide information on drugs/products and services and answer questions as part of therapeutic regimens associated with a pathology linked to the concerned drugs | Very Confident | 230 | 64.35% (148) |
|  | Fairly confident |  | 32.61% (75) |
|  | Neither/I don’t know |  | 0.87% (2) |
|  | Slightly confident |  | 2.17% (5) |
|  | Not confident at all |  | 0 |
| **PK3 =** Answer questions of healthcare professionals on drugs/products and services as part of comprehensive patient care | Very confident | 230 | 69.56% (160) |
|  | Fairly confident |  | 28.70% (66) |
|  | Neither/I don’t know |  | 0.87% (2) |
|  | Slightly confident |  | 0.87% (2) |
|  | Not confident at all |  | 0 |
| **PK4 =** Link scientific and medical knowledge to drug/product arguments | Very confident | 230 | 75.22% (173) |
|  | Fairly confident |  | 22.61% (52) |
|  | Neither/I don’t know |  | 0.87% (2) |
|  | Slightly confident |  | 1.30% (3) |
|  | Not confident at all |  | 0 |
| **PK5 =** Exchange with healthcare professionals on scientific topics | Very confident | 230 | 73.04% (168) |
|  | Fairly confident |  | 23.48% (54) |
|  | Neither/I don’t know |  | 1.74% (4) |
|  | Slightly confident |  | 1.30% (3) |
|  | Not confident at all |  | 0.44% (1) |
| **PK6 =** Maintain and develop product knowledge through training | Very confident | 230 | 74.78% (172) |
|  | Fairly confident |  | 22.61% (52) |
|  | Neither/I don’t know |  | 0.87% (2) |
|  | Slightly confident |  | 1.30% (3) |
|  | Not confident at all |  | 0.44% (1) |
| **PCS = Professional Communication Skills** | | | |
| **PCSC = Communication** | | | |
| **PCSC1 =** Display knowledge of pharmaceuticals during sales presentations to doctors and other healthcare professionals | Very confident | 230 | 81.30% (187) |
|  | Fairly confident |  | 18.26% (42) |
|  | Neither/I don’t know |  | 0 |
|  | Slightly confident |  | 0 |
|  | Not confident at all |  | 0.44% (1) |
| **PCSC2 =** Summarize the key elements involved in medical/marketing communication in the healthcare environment | Very Confident | 230 | 80.87% (186) |
|  | Fairly confident |  | 17.83% (41) |
|  | Neither/I don’t know |  | 0.44% (1) |
|  | Slightly confident |  | 0.44% (1) |
|  | Not confident at all |  | 0.44% (1) |
| **PCSC3 =** Explain the characteristics and the proper use of drugs/products based on the needs of healthcare professionals and market demands | Very confident | 230 | 83.03% (191) |
|  | Fairly confident |  | 15.22% (35) |
|  | Neither/I don’t know |  | 0.87% (2) |
|  | Slightly confident |  | 0.44% (1) |
|  | Not confident at all |  | 0.44% (1) |
| **PCSC4 =** Take ownership of the content of the information prepared by the scientists responsible for the pharmaceutical company | Very confident | 230 | 67.39% (155) |
|  | Fairly confident |  | 28.70% (66) |
|  | Neither/I don’t know |  | 1.74% (4) |
|  | Slightly confident% |  | 0.87(2) |
|  | Not confident at all |  | 1.30% (3) |
| **PCSC5 =** Use the information, arguments, business aids developed by the pharmaceutical company | Very confident | 230 | 81.73% (188) |
|  | Fairly confident |  | 16.09% (37) |
|  | Neither/I don’t know |  | 1.74% (4) |
|  | Slightly confident |  | 0 |
|  | Not confident at all |  | 0.44% (1) |
| **PCSC6 =** Use effective verbal, non-verbal, listening, and written communication skills to communicate accurately and appropriately | Very confident | 230 | 81.31% (187) |
|  | Fairly confident |  | 15.65% (36) |
|  | Neither/I don’t know |  | 1.74% (4) |
|  | Slightly confident |  | 1.30% (3) |
|  | Not confident at all |  | 0 |
| **PCSC7 =** Communicate effectively with physicians, other healthcare professionals, support staff, and relevant third parties | Very confident | 230 | 83.04% (191) |
|  | Fairly confident |  | 14.78% (34) |
|  | Neither/I don’t know |  | 1.30% (3) |
|  | Slightly confident |  | 0.44% (1) |
|  | Not confident at all |  | 0.44% (1) |
| **PCSC8 =** Use appropriate language and checks comprehension | Very confident | 230 | 82.60% (190) |
|  | Fairly confident |  | 14.78% (34) |
|  | Neither/I don’t know |  | 1.74% (4) |
|  | Slightly confident |  | 0.44% (1) |
|  | Not confident at all |  | 0.44% (1) |
| **PCSC9 =** Demonstrate respect, cultural awareness, sensitivity, and empathy when communicating | Very confident | 230 | 85.65% (197) |
|  | Fairly confident |  | 12.17% (28) |
|  | Neither/I don’t know |  | 1.30% (3) |
|  | Slightly confident |  | 0.44% (1) |
|  | Not confident at all |  | 0.44% (1) |
| **PCSN = Negotiation** | | | |
| **PCSN1 =** Establish a quality relationship with healthcare professionals | Very confident | 230 | 87.39% (201) |
|  | Fairly confident |  | 11.74% (27) |
|  | Neither/I don’t know |  | 0.44% (1) |
|  | Slightly confident |  | 0 |
|  | Not confident at all |  | 0.44% (1) |
| **PCSN2 =** Identify/address the healthcare professionals’ concerns/needs and their patient care practices by using appropriate probing/questioning | Very Confident | 230 | 83.47% (192) |
|  | Fairly confident |  | 15.21% (35) |
|  | Neither/I don’t know |  | 0.44% (1) |
|  | Slightly confident |  | 0.44% (1) |
|  | Not confident at all |  | 0.44% (1) |
| **PCSN3 =** Apply active listening techniques with the healthcare professional | Very confident | 230 | 86.52% (199) |
|  | Fairly confident |  | 12.17% (28) |
|  | Neither/I don’t know |  | 0.44% (1) |
|  | Slightly confident |  | 0 |
|  | Not confident at all |  | 0.87% (2) |
| **PCSN4 =** Demonstrate knowledge of sales techniques | Very confident | 230 | 80.43% (185) |
|  | Fairly confident |  | 18.26% (42) |
|  | Neither/I don’t know |  | 0.44% (1) |
|  | Slightly confident% |  | 0 |
|  | Not confident at all |  | 0.87% (2) |
| **PCSN5 =** Adapt to different communication styles | Very confident | 230 | 82.17% (189) |
|  | Fairly confident |  | 17.39% (40) |
|  | Neither/I don’t know |  | 0 |
|  | Slightly confident |  | 0 |
|  | Not confident at all |  | 0.44% (1) |
| **PCSN6 =** Process requests for information and objections | Very confident | 230 | 81.31% (187) |
|  | Fairly confident |  | 15.65% (36) |
|  | Neither/I don’t know |  | 1.74% (4) |
|  | Slightly confident |  | 1.30% (3) |
|  | Not confident at all |  | 0 |
| **PCSN7 =** Conclude the call/visit & prepare reports | Very confident | 230 | 81.73% (188) |
|  | Fairly confident |  | 17.83% (41) |
|  | Neither/I don’t know |  | 0 |
|  | Slightly confident |  | 0 |
|  | Not confident at all |  | 0.44% (1) |
| **PCSN8 =** Analyze the call/visit (SWOC analysis) and plan the next step | Very confident | 230 | 70.00% (161) |
|  | Fairly confident |  | 27.39% (63) |
|  | Neither/I don’t know |  | 2.17% (5) |
|  | Slightly confident |  | 0 |
|  | Not confident at all |  | 0.44% (1) |
| **PCSN9 =** Animate professional communication gatherings and develop long-term professional relationships/partnerships with healthcare professionals | Very confident | 230 | 75.65% (174) |
|  | Fairly confident |  | 22.61% (52) |
|  | Neither/I don’t know |  | 1.30% (3) |
|  | Slightly confident |  | 0 |
|  | Not confident at all |  | 0.44% (1) |
| **PCSD = Data Processing Analysis Skills** | | | |
| **PCSD1 =** Collect and process information on drugs/products, from documentation and training sessions to prepare for visits and communication actions | Very confident | 230 | 70.87% (163) |
|  | Fairly confident |  | 26.09% (60) |
|  | Neither/I don’t know |  | 3.04% (7) |
|  | Slightly confident |  | 0 |
|  | Not confident at all |  | 0 |
| **PCSD2 =** Collect, analyze and transmit questions to the concerned departments of the company | Very Confident | 230 | 70.87% (163) |
|  | Fairly confident |  | 27.39% (63) |
|  | Neither/I don’t know |  | 1.74% (4) |
|  | Slightly confident |  | 0 |
|  | Not confident at all |  | 0 |
| **PCSD3 =** Collect and transmit pharmacovigilance information | Very confident | 230 | 63.04% (145) |
|  | Fairly confident |  | 32.17% (74) |
|  | Neither/I don’t know |  | 4.35% (10) |
|  | Slightly confident |  | 0.44% (1) |
|  | Not confident at all |  | 0 |
| **PCSD4 =** Describe the commercial healthcare environment in which pharmaceutical medicine operates | Very confident | 230 | 69.56% (160) |
|  | Fairly confident |  | 26.52% (61) |
|  | Neither/I don’t know |  | 2.62% (6) |
|  | Slightly confident% |  | 1.30% (3) |
|  | Not confident at all |  | 0 |
| **PCSD5 =** Appraise the commercial competitor environment when evaluating the opportunity for new medicine under development or a currently marketed product | Very confident | 230 | 66.52% (153) |
|  | Fairly confident |  | 29.57% (68) |
|  | Neither/I don’t know |  | 3.04% (7) |
|  | Slightly confident |  | 0.87% (2) |
|  | Not confident at all |  | 0 |
| **PCSD6 =** Apply competitive intelligence and report information to its hierarchy | Very confident | 230 | 68.70% (158) |
|  | Fairly confident |  | 27.82% (64) |
|  | Neither/I don’t know |  | 3.04% (7) |
|  | Slightly confident |  | 0.44% (1) |
|  | Not confident at all |  | 0 |
| **PCSD7 =** Monitor actions and professional communication during visits | Very confident | 230 | 76.09% (175) |
|  | Fairly confident |  | 21.74% (50) |
|  | Neither/I don’t know |  | 1.30% (3) |
|  | Slightly confident |  | 0.87% (2) |
|  | Not confident at all |  | 0 |
| **PCSI = Information Technology** | | | |
| **PCSI1 =** Save and transmit calls/visits reports to the company database | Very confident | 230 | 79.56 (183) |
|  | Fairly confident |  | 18.27% (42) |
|  | Neither/I don’t know |  | 2.17% (5) |
|  | Slightly confident |  | 0 |
|  | Not confident at all |  | 0 |
| **PCSI2 =** Inform and update files | Very Confident | 230 | 76.96% (177) |
|  | Fairly confident |  | 21.30% (49) |
|  | Neither/I don’t know |  | 1.30% (3) |
|  | Slightly confident |  | 0.44% (1) |
|  | Not confident at all |  | 0 |
| **PCSI3 =** Master research of information via electronic databases | Very confident | 230 | 63.04% (145) |
|  | Fairly confident |  | 32.17% (74) |
|  | Neither/I don’t know |  | 4.35% (10) |
|  | Slightly confident |  | 0.44% (1) |
|  | Not confident at all |  | 0 |
| **PCSI4 =** Optimize the use of computerized/electronic devices to prepare presentations, reports, charts, and manage business and information processing | Very confident | 230 | 69.56% (160) |
|  | Fairly confident |  | 26.52% (61) |
|  | Neither/I don’t know |  | 2.62% (6) |
|  | Slightly confident% |  | 1.30% (3) |
|  | Not confident at all |  | 0 |
| **PCSSMS = Self-Management Skills** | | | |
| **PCSSMS1 =** Demonstrate organization and efficiency in carrying out the work | Very confident | 230 | 78.70% (181) |
|  | Fairly confident |  | 20.00% (46) |
|  | Neither/I don’t know |  | 0.87% (2) |
|  | Slightly confident |  | 0.43% (1) |
|  | Not confident at all |  | 0 |
| **PCSSMS2 =** Organize visits according to the predefined objectives and through teamwork | Very Confident | 230 | 76.96% (177) |
|  | Fairly confident |  | 20.43% (47) |
|  | Neither/I don’t know |  | 1.74% (4) |
|  | Slightly confident |  | 0.87% (2) |
|  | Not confident at all |  | 0 |
| **PCSSMS3 =** Ensure work time and processes are planned and managed appropriately | Very confident | 230 | 77.83% (179) |
|  | Fairly confident |  | 20.43% (47) |
|  | Neither/I don’t know |  | 1.74% (4) |
|  | Slightly confident |  | 0 |
|  | Not confident at all |  | 0 |
| **PCSSMS4 =** Demonstrate the ability to prioritize work appropriately | Very confident | 230 | 80.00% (184) |
|  | Fairly confident |  | 18.70% (43) |
|  | Neither/I don’t know |  | 1.30% (3) |
|  | Slightly confident% |  | 0 |
|  | Not confident at all |  | 0 |
| **PCSSMS5 =** Take responsibility as appropriate in the workplace | Very confident | 230 | 82.61% (190) |
|  | Fairly confident |  | 16.52% (38) |
|  | Neither/I don’t know |  | 0.87% (2) |
|  | Slightly confident |  | 0 |
|  | Not confident at all |  | 0 |
| **PCSSMS6 =** Ensure punctuality and reliability | Very confident | 230 | 80.43% (185) |
|  | Fairly confident |  | 17.83% (41) |
|  | Neither/I don’t know |  | 1.30% (3) |
|  | Slightly confident |  | 0.44% (1) |
|  | Not confident at all |  | 0 |
| **PCSSMS7 =** Reflect on and demonstrate learning from critical incidents | Very confident | 230 | 81.30% (187) |
|  | Fairly confident |  | 16.96% (39) |
|  | Neither/I don’t know |  | 1.74% (4) |
|  | Slightly confident |  | 0 |
|  | Not confident at all |  | 0 |
| **PCSSMS8 =** Engage in regular professional development activities | Very confident | 230 | 75.65% (174) |
|  | Fairly confident |  | 21.74% (50) |
|  | Neither/I don’t know |  | 2.61% (6) |
|  | Slightly confident |  | 0 |
|  | Not confident at all |  | 0 |
| **PCSSMS9 =** Engage in professional organization activities | Very confident | 230 | 78.70% (181) |
|  | Fairly confident |  | 18.26% (42) |
|  | Neither/I don’t know |  | 3.04% (7) |
|  | Slightly confident |  | 0 |
|  | Not confident at all |  | 0 |
| **PCSMS = Management Skills** | | | |
| **PCSMS1 =** Describe the pharmaceutical industry (internal environment, structure and function, key stakeholders and commercial drivers) and explain how these business elements impact on the broader healthcare market place | Very confident | 230 | 60.00% (138) |
|  | Fairly confident |  | 33.04% (76) |
|  | Neither/I don’t know |  | 4.35% (10) |
|  | Slightly confident |  | 2.61% (6) |
|  | Not confident at all |  | 0 |
| **PCSMS2 =** Demonstrate an understanding of the principles of organization and management | Very Confident | 230 | 60.87% (140) |
|  | Fairly confident |  | 35.65% (82) |
|  | Neither/I don’t know |  | 2.17% (5) |
|  | Slightly confident |  | 1.30% (3) |
|  | Not confident at all |  | 0 |
| **PCSMS3 =** Work effectively with the documented procedures and policies within the workplace | Very confident | 230 | 71.31% (164) |
|  | Fairly confident |  | 25.65% (59) |
|  | Neither/I don’t know |  | 1.74% (4) |
|  | Slightly confident |  | 1.30% (3) |
|  | Not confident at all |  | 0 |
| **PCSMS4 =** Work effectively with the company hierarchy | Very confident | 230 | 77.83% (179) |
|  | Fairly confident |  | 20.00% (46) |
|  | Neither/I don’t know |  | 0.87% (2) |
|  | Slightly confident% |  | 1.30% (3) |
|  | Not confident at all |  | 0 |
| **PCSMS5 =** Provide regular feedback on the drugs/products and the market | Very confident | 230 | 75.22% (173) |
|  | Fairly confident |  | 23.48% (54) |
|  | Neither/I don’t know |  | 0.44% (1) |
|  | Slightly confident |  | 0.87% (2) |
|  | Not confident at all |  | 0 |
| **PCSMS6 =** Convey any helpful information from the market with all the company's concerned people/departments (medical representatives, direct manager, product manager, medical manager, medical science liaison, CRA, etc) | Very confident | 230 | 73.91% (170) |
|  | Fairly confident |  | 23.48% (54) |
|  | Neither/I don’t know |  | 2.17% (5) |
|  | Slightly confident |  | 0.44% (1) |
|  | Not confident at all |  | 0 |
| **PCSMS7 =** Apply the company's compliance, procedures, and safety rules (road, IT, etc) | Very confident | 230 | 76.09% (175) |
|  | Fairly confident |  | 20.00% (46) |
|  | Neither/I don’t know |  | 2.17% (5) |
|  | Slightly confident |  | 1.74% (4) |
|  | Not confident at all |  | 0 |
| **PCSMS8 =** Organize round tables, expert meetings, advisory boards, lectures, CME conferences, staff meetings, awareness campaigns, and others in coordination with different departments within the company and service providers | Very confident | 230 | 70.87% (163) |
|  | Fairly confident |  | 23.91% (55) |
|  | Neither/I don’t know |  | 3.91% (9) |
|  | Slightly confident |  | 0.87% (2) |
|  | Not confident at all |  | 0.44% (1) |
| **PCSMS9 =** Apply the national and international code of ethics guidelines when organizing any of the scientific events mentioned above | Very confident | 230 | 72.18% (166) |
|  | Fairly confident |  | 22.61% (52) |
|  | Neither/I don’t know |  | 3.91% (9) |
|  | Slightly confident |  | 1.30% (3) |
|  | Not confident at all |  | 0 |
| **PCSP = Standard Practice** | | | |
| **PCSP1** = Carry out duties as a medical representative in a professional manner | Very confident | 230 | 85.65% (197) |
|  | Fairly confident |  | 12.61% (29) |
|  | Neither/I don’t know |  | 1.30% (3) |
|  | Slightly confident |  | 0.44% (1) |
|  | Not confident at all |  | 0 |
| **PCSP2** = Demonstrate awareness of the position of trust of the profession and practice in a manner that upholds that trust | Very Confident | 230 | 81.74% (188) |
|  | Fairly confident |  | 16.96% (39) |
|  | Neither/I don’t know |  | 1.30% (3) |
|  | Slightly confident |  | 0 |
|  | Not confident at all |  | 0 |
| **PCSP3** = Treat others with sensitivity, empathy, respect, and dignity | Very confident | 230 | 85.22% (193) |
|  | Fairly confident |  | 13.91% (32) |
|  | Neither/I don’t know |  | 0.87% (2) |
|  | Slightly confident |  | 0 |
|  | Not confident at all |  | 0 |
| **PCSP4** = Take responsibility for their own actions | Very confident | 230 | 82.18% (189) |
|  | Fairly confident |  | 15.65% (36) |
|  | Neither/I don’t know |  | 1.30% (3) |
|  | Slightly confident% |  | 0.87% (2) |
|  | Not confident at all |  | 0 |
| **PCSP5** = Recognize their scope of practice and the extent of their current competency and expertise and works accordingly | Very confident | 230 | 75.65% (174) |
|  | Fairly confident |  | 21.74% (50) |
|  | Neither/I don’t know |  | 2.17% (5) |
|  | Slightly confident |  | 0.44% (1) |
|  | Not confident at all |  | 0 |
| **PCSP6** = Maintain a consistently high standard of work | Very confident | 230 | 85.65% (197) |
|  | Fairly confident |  | 12.61% (29) |
|  | Neither/I don’t know |  | 1.30% (3) |
|  | Slightly confident |  | 0.44% (1) |
|  | Not confident at all |  | 0 |
| **PCSE = Ethical Practice** | | | |
| **PCSE1** = Understand obligations under the principles of the statutory Code of Conduct for Pharmacists and act accordingly | Very confident | 230 | 80.43% (185) |
|  | Fairly confident |  | 18.27% (42) |
|  | Neither/I don’t know |  | 1.30% (3) |
|  | Slightly confident |  | 0 |
|  | Not confident at all |  | 0 |
| **PCSE2** = Make and justify decisions in a manner that reflects the statutory Code of Conduct for pharmacists and pharmacy law | Very Confident | 230 | 76.53% (176) |
|  | Fairly confident |  | 21.30% (49) |
|  | Neither/I don’t know |  | 2.17% (5) |
|  | Slightly confident |  | 0 |
|  | Not confident at all |  | 0 |
| **PCSE3** = Recognize ethical dilemmas in practice scenarios and reason through dilemmas in a structured manner | Very confident | 230 | 76.96% (177) |
|  | Fairly confident |  | 20.43% (47) |
|  | Neither/I don’t know |  | 2.17% (5) |
|  | Slightly confident |  | 0.44% (1) |
|  | Not confident at all |  | 0 |
| **PCSE4** = Implement standard operating procedures and Code of Ethics | Very confident | 230 | 78.26% (180) |
|  | Fairly confident |  | 20.87% (48) |
|  | Neither/I don’t know |  | 0.87% (2) |
|  | Slightly confident% |  | 0 |
|  | Not confident at all |  | 0 |
| **PCSL = Legal Practice** | | | |
| **PCSL1** = Identify laws and regulations related to sales and marketing practices | Very confident | 230 | 67.83% (156) |
|  | Fairly confident |  | 28.26% (65) |
|  | Neither/I don’t know |  | 3.04% (7) |
|  | Slightly confident |  | 0.87% (2) |
|  | Not confident at all |  | 0 |
| **PCSL2** = Demonstrate an awareness of and adheres to professional indemnity requirements | Very Confident | 230 | 70.87% (163) |
|  | Fairly confident |  | 24.78% (57) |
|  | Neither/I don’t know |  | 2.61% (6) |
|  | Slightly confident |  | 1.74% (4) |
|  | Not confident at all |  | 0 |
| **PCSL3** = Use and take into account the drug-related pharmaceutical and economic regulation and its evolution to inform and answer questions from healthcare professionals | Very confident | 230 | 71.74% (165) |
|  | Fairly confident |  | 24.78% (57) |
|  | Neither/I don’t know |  | 3.04% (7) |
|  | Slightly confident |  | 0.44% (1) |
|  | Not confident at all |  | 0 |
| **PCSL4** = Integrate into business the rules of advertising, promotion, distribution, and delivery of the drug and their changes | Very confident | 230 | 72.17% (166) |
|  | Fairly confident |  | 23.04% (53) |
|  | Neither/I don’t know |  | 3.48% (8) |
|  | Slightly confident% |  | 0.87% (2) |
|  | Not confident at all |  | 0.44% (1) |
| **PCSL5** = Raise awareness and provide information on regulatory changes | Very confident | 230 | 67.83% (156) |
|  | Fairly confident |  | 26.52% (61) |
|  | Neither/I don’t know |  | 4.34% (10) |
|  | Slightly confident |  | 0.87% (2) |
|  | Not confident at all |  | 0.44% (1) |
| **PCSL6** = Use tools related to the product (summary of product characteristics, product file, transparency commission opinion, validated data, etc) | Very confident | 230 | 75.22% (173) |
|  | Fairly confident |  | 21.74% (50) |
|  | Neither/I don’t know |  | 1.74% (4) |
|  | Slightly confident |  | 1.30% (3) |
|  | Not confident at all |  | 0 |
| **PCSL7** = Drive up pharmacovigilance information by following the internal procedures and regulations | Very confident | 230 | 65.65% (151) |
|  | Fairly confident |  | 27.39% (63) |
|  | Neither/I don’t know |  | 4.35% (10) |
|  | Slightly confident |  | 2.61% (6) |
|  | Not confident at all |  | 0 |
| **PCSR = Role Modeling** | | | |
| **PCSR1** = Inspire confidence and apply assertiveness skills as appropriate | Very confident | 230 | 79.56% (183) |
|  | Fairly confident |  | 19.13% (44) |
|  | Neither/I don’t know |  | 0.44% (1) |
|  | Slightly confident |  | 0.87% (2) |
|  | Not confident at all |  | 0 |
| **PCSR2** = Build credibility and portray the profession in a positive light by being professional and well-informed | Very Confident | 230 | 70.87% (163) |
|  | Fairly confident |  | 24.78% (57) |
|  | Neither/I don’t know |  | 2.61% (6) |
|  | Slightly confident |  | 1.74% (4) |
|  | Not confident at all |  | 0 |
| **PCSR3** = Contribute to the initiation, development, and continuous improvement of business plans | Very confident | 230 | 81.30% (187) |
|  | Fairly confident |  | 17.83% (41) |
|  | Neither/I don’t know |  | 0 |
|  | Slightly confident |  | 0.87% (2) |
|  | Not confident at all |  | 0 |
| **PCSR4** = Have effective leadership skills | Very confident | 230 | 75.22% (173) |
|  | Fairly confident |  | 22.17% (51) |
|  | Neither/I don’t know |  | 1.74% (4) |
|  | Slightly confident% |  | 0.87% (2) |
|  | Not confident at all |  | 0 |
| **PCST = Team Working Skills** | | | |
| **PCST1** = Recognize the value of transversal teamwork | Very confident | 230 | 83.04% (191) |
|  | Fairly confident |  | 13.91% (32) |
|  | Neither/I don’t know |  | 2.17% (5) |
|  | Slightly confident |  | 0.44% (1) |
|  | Not confident at all |  | 0.44% (1) |
| **PCST2** = Recognize when it is appropriate to seek advice from experienced colleagues, refer decisions to a higher level of authority, or include other colleagues in the decision | Very Confident | 230 | 83.48% (192) |
|  | Fairly confident |  | 15.65% (36) |
|  | Neither/I don’t know |  | 0.87% (2) |
|  | Slightly confident |  | 0 |
|  | Not confident at all |  | 0 |
| **PP = Pharmacist Preparedness and Response in Emergency Situations** | | | |
| **PPE = Emergency Preparedness and Response** | | | |
| **PPE1** = Check for volunteering opportunities | Very confident | 230 | 47.82% (110) |
|  | Fairly confident |  | 30.00% (69) |
|  | Neither/I don’t know |  | 16.09% (37) |
|  | Slightly confident |  | 4.35% (10) |
|  | Not confident at all |  | 1.74% (4) |
| **PPE2** = Check for training opportunities | Very Confident | 230 | 58.70% (135) |
|  | Fairly confident |  | 33.36% (77) |
|  | Neither/I don’t know |  | 5.66% (13) |
|  | Slightly confident |  | 1.30% (3) |
|  | Not confident at all |  | 0.88% (2) |
| **PPE3** = Address medication shortage and mitigation plan | Very confident | 230 | 56.96% (131) |
|  | Fairly confident |  | 30.43% (70) |
|  | Neither/I don’t know |  | 8.70% (20) |
|  | Slightly confident |  | 3.47% (8) |
|  | Not confident at all |  | 0.44% (1) |
| **PPE4** = Balance stockpile and availability of drugs for existing/chronic conditions | Very confident | 230 | 54.37% (125) |
|  | Fairly confident |  | 29.55% (68) |
|  | Neither/I don’t know |  | 11.73% (27) |
|  | Slightly confident% |  | 3.47(8) |
|  | Not confident at all |  | 0.88% (2) |
| **PPE5** = Partner with local authorities | Very confident | 230 | 43.48% (100) |
|  | Fairly confident |  | 36.52% (84) |
|  | Neither/I don’t know |  | 16.52% (38) |
|  | Slightly confident |  | 3.04% (7) |
|  | Not confident at all |  | 0.44% (1) |
| **PPE6** = Check for FDA/EMA Emergency Use Authorizations (EUAs) and expedited review and approval of tests/drugs for treatment | Very confident | 230 | 53.04% (122) |
|  | Fairly confident |  | 33.48% (77) |
|  | Neither/I don’t know |  | 10.87% (25) |
|  | Slightly confident |  | 2.17% (5) |
|  | Not confident at all |  | 0.44% (1) |
| **PPE7** = Follow actions and recommendations of local authorities | Very confident | 230 | 66.09% (152) |
|  | Fairly confident |  | 24.78% (57) |
|  | Neither/I don’t know |  | 6.52% (15) |
|  | Slightly confident |  | 2.61% (6) |
|  | Not confident at all |  | 0 |
| **PPO = Operation Management** | | | |
| **PPO1** = Procure essential medications and supplies | Very confident | 230 | 51.74% (119) |
|  | Fairly confident |  | 31.30% (72) |
|  | Neither/I don’t know |  | 13.04% (30) |
|  | Slightly confident |  | 3.48% (8) |
|  | Not confident at all |  | 0.44% (1) |
| **PPO2** = Ensure medication delivery/safe storage | Very Confident | 230 | 58.26% (134) |
|  | Fairly confident |  | 29.56% (68) |
|  | Neither/I don’t know |  | 10% (23) |
|  | Slightly confident |  | 1.74% (4) |
|  | Not confident at all |  | 0.44% (1) |
| **PPO3** = Develop workplace training and safety protocols (e.g., social distancing) | Very confident | 230 | 60.00% (138) |
|  | Fairly confident |  | 25.65% (59) |
|  | Neither/I don’t know |  | 11.30% (26) |
|  | Slightly confident |  | 2.18% (5) |
|  | Not confident at all |  | 0.87% (2) |
| **PPO4** = Secure PPEs or other needed materials | Very confident | 230 | 52.18% (120) |
|  | Fairly confident |  | 26.52% (61) |
|  | Neither/I don’t know |  | 17.39% (40) |
|  | Slightly confident% |  | 2.61% (6) |
|  | Not confident at all |  | 1.30% (3) |
| **PPO5** = Monitor workers/assistants for symptoms | Very confident | 230 | 50.87% (117) |
|  | Fairly confident |  | 27.39% (63) |
|  | Neither/I don’t know |  | 17.83% (41) |
|  | Slightly confident |  | 3.47% (8) |
|  | Not confident at all |  | 0.44% (1) |
| **PPO6** = Adapt working hours to meet essential services during crises | Very confident | 230 | 55.22% (127) |
|  | Fairly confident |  | 31.74% (73) |
|  | Neither/I don’t know |  | 10.87% (25) |
|  | Slightly confident |  | 1.30% (3) |
|  | Not confident at all |  | 0.87% (2) |
| **PPO7** = Secure sanitizers and other medications when needed | Very confident | 230 | 56.96% (131) |
|  | Fairly confident |  | 29.13% (67) |
|  | Neither/I don’t know |  | 11.74% (27) |
|  | Slightly confident |  | 1.30% (3) |
|  | Not confident at all |  | 0.87% (2) |
| **PPO8** = Participate in interdisciplinary training to EPR teams | Very confident | 230 | 47.84% (110) |
|  | Fairly confident |  | 29.56% (68) |
|  | Neither/I don’t know |  | 19.13% (44) |
|  | Slightly confident |  | 2.17% (5) |
|  | Not confident at all |  | 1.30% (3) |
| **PPC = Patient Care and Population Health Interventions** | | | |
| **PPC1** = Manage panic buying | Very confident | 230 | 31.74% (73) |
|  | Fairly confident |  | 40.43% (93) |
|  | Neither/I don’t know |  | 22.18% (51) |
|  | Slightly confident |  | 4.35% (10) |
|  | Not confident at all |  | 1.30% (3) |
| **PPC2** = Answer EPR-related calls | Very Confident | 230 | 30.87% (71) |
|  | Fairly confident |  | 36.53% (84) |
|  | Neither/I don’t know |  | 27.39% (63) |
|  | Slightly confident |  | 3.91% (9) |
|  | Not confident at all |  | 1.30% (3) |
| **PPERD = Evaluation, Research, and Dissemination for Impact and Outcomes** | | | |
| **PPERD1** = Participate in research and studies on EPR | Very confident | 230 | 25.22% (58) |
|  | Fairly confident |  | 34.78% (80) |
|  | Neither/I don’t know |  | 34.35(79) |
|  | Slightly confident |  | 2.17% (5) |
|  | Not confident at all |  | 3.48% (8) |
| **PPERD2** = Publish and/or disseminate findings | Very Confident | 230 | 23.48% (54) |
|  | Fairly confident |  | 33.48% (77) |
|  | Neither/I don’t know |  | 35.65% (82) |
|  | Slightly confident |  | 2.17% (5) |
|  | Not confident at all |  | 5.22% (12) |
| **PPERD3** = Combat misinformation by disseminating evidence-based information to patients and sharing it on social media | Very confident | 230 | 30.43% (70) |
|  | Fairly confident |  | 35.22% (81) |
|  | Neither/I don’t know |  | 27.39% (63) |
|  | Slightly confident |  | 3.91% (9) |
|  | Not confident at all |  | 3.04% (7) |
| **PPERD4** = Develop training programs for peers and other healthcare workers | Very confident | 230 | 33.91% (78) |
|  | Fairly confident |  | 33.48% (77) |
|  | Neither/I don’t know |  | 25.65% (59) |
|  | Slightly confident |  | 3.04% (7) |
|  | Not confident at all |  | 3.91% (9) |

| Variable | Subgroups |  | N | n (%) | Median | Min-Max | Mean-SD |
| --- | --- | --- | --- | --- | --- | --- | --- |
|  | **Percentage of competencies acquired by the pharmacist** | | | | | |  |
|  | **During undergraduate studies** | | | | | |  |
| **CompUnderGrad1** | 0-25% |  | 230 | 46.52% (107) | 30 | 0-100 | 33.25-24.26 |
|  | 26-50% |  |  | 34.35% (79) |  |  |  |
|  | 51-75% |  |  | 12.61% (29) |  |  |  |
|  | 76-100% |  |  | 6.52% (15) |  |  |  |
|  | **During postgraduate studies** | | | | | |  |
| **CompPostGrad1** | 0-25% |  | 230 | 47.39% (109) | 30 | 0-100 | 37.39-31.84 |
|  | 26-50% |  |  | 20% (46) |  |  |  |
|  | 51-75% |  |  | 13.48% (31) |  |  |  |
|  | 76-100% |  |  | 19.13% (44) |  |  |  |
|  | **From continuing education sessions** | | | | | |  |
| **CompCE1** | 0-25% |  | 230 | 54.78% (126) | 20 | 0-100 | 33.14-30.67 |
|  | 26-50% |  |  | 21.74% (50) |  |  |  |
|  | 51-75% |  |  | 8.70% (20) |  |  |  |
|  | 76-100% |  |  | 14.78% (34) |  |  |  |
|  | **By experience** | | | | | |  |
| **CompExp1** | 0-25% |  | 230 | 4.78% (11) | 70 | 3-100 | 69.06-23.71 |
|  | 26-50% |  |  | 28.26% (65) |  |  |  |
|  | 51-75% |  |  | 20.00% (46) |  |  |  |
|  | 76-100% |  |  | 46.96% (108) |  |  |  |

**Table S3:** Description of the specialized competencies of sales and marketing pharmacists

| **Scale** | **Subscale** | **Mean** | **Median** | **Standard Deviation** | **Upper Value** | **Theoretical Upper value** | **Standardized mean (/100)** |
| --- | --- | --- | --- | --- | --- | --- | --- |
| **Pharmaceutical Knowledge (PK)** | - | 24.95 | 26 | 2.89 | 27 | 30 | 83.17 |
| **Professional Communication Skills (PCS)** | Communication | 38.96 | 41 | 3.71 | 41 | 45 | 86.58 |
|  | Negotiation | 32.15 | 34 | 3.09 | 34 | 45 | 71.44 |
|  | Data Processing Analysis Skills | 23.59 | 25 | 3.07 | 26 | 35 | 67.40 |
|  | Information Technology | 13.78 | 15 | 1.79 | 15 | 20 | 68.90 |
|  | Self-Management Skills | 27.93 | 30 | 3.37 | 30 | 45 | 62.07 |
|  | Management Skills | 33.92 | 35 | 3.99 | 37 | 45 | 75.38 |
|  | Standard Practice | 20.83 | 22 | 2.25 | 22 | 30 | 69.43 |
|  | Ethical Practice | 12.05 | 13 | 1.56 | 13 | 20 | 60.25 |
|  | Legal Practice | 27.49 | 29 | 3.48 | 30 | 35 | 78.54 |
|  | Role Modeling | 14 | 15 | 1.62 | 15 | 20 | 70.00 |
|  | Team Work Skills | 7.61 | 8 | 0.85 | 8 | 10 | 76.10 |
| **Pharmacist Preparedness** **and Response in Emergency Situations (PP)** | Emergency Preparedness and Response | 29.49 | 32 | 4.61 | 35 | 35 | 84.25 |
|  | Operation Management | 34.66 | 36 | 5.99 | 40 | 40 | 86.65 |
|  | Patient Care and Population Health Interventions | 7.89 | 8 | 1.75 | 10 | 10 | 78.90 |
|  | Evaluation, Research, and Dissemination for Impact and Outcomes | 15.2 | 16 | 3.65 | 20 | 20 | 76.00 |

**Table S4:** Bivariate Analysis *– Correlates of Pharmaceutical Knowledge*

| **Pharmaceutical Knowledge** | |
| --- | --- |
| **Variable** | **p-value**  **Mean (sd)** |
| **Gender**  Male=65  Female=165 | **0.855**  24.89 (3.50)  24.97 (2.62) |
| **BS**  Yes=196  No=34 | **0.512**  25.00 (2.87)  24.65 (2.91) |
| **PharmD/DPharm**  Yes=92  No=138 | **0.738**  25.00 (2.60)  24.87 (3.07) |
| **Master**  Yes=72  No=158 | **0.548**  24.78 (3.08)  25.02 (2.80) |
| **PhD**  Yes=8  No=222 | **0.775**  -  - |
| **HighDeg**  BS=94  PharmD/DPharm=34  Master=52  PhD or equivalent=6  Others=4 | **0.017***  25.23 (2.78)  24.80 (2.72)  24.94 (2.52)  25.50 (2.34)  20.25 (8.06) |
| **YearGrad1**  1989-1999=14  2000-2010=63  2011-2020=153 | **0.189**  24.93 (2.87)  25.51 (2.57)  24.72 (3.00) |
| **UniPh**  Lebanese American University=46  Lebanese International University=46  Saint Joseph University of Beirut=46  Beirut Arab University=39  Lebanese University=32  Foreign University=18  NA=3 | **0.385**  25.33 (3.12)  24.28 (3.54)  24.61 (2.90)  25.36 (2.07)  25.00 (2.69)  25.50 (2.25) |
| **UniHighDeg**  Lebanese American University=38  Lebanese International University=37  Saint Joseph University of Beirut=45  Beirut Arab University=30  Lebanese University=40  OtherLeb=19  OtherForeign=19  NA=2 | **0.411**  25.53 (2.05)  24.24 (3.76)  24.84 (2.88)  25.23 (2.11)  25.05 (2.51)  24.37 (4.46)  25.68 (1.97) |
| **Lang**  English=146  French=78  Other=6 | **0.626**  25.03 (2.98)  24.73 (2.75)  25.67 (2.42) |
| **WLoc**  Beirut=121  Beqaa=12  Mount Lebanon=44  North Lebanon=29  South Lebanon=8  Currently not working=16 | **0.834**  24.77 (3.16)  24.42 (3.42)  25.16 (2.54)  25.21 (2.53)  25.75 (1.64)  25.25 (2.52) |
| **Wdays/wk**  0=5  2=1  3=2  5=214  6=8 | **0.323**  24.4 (2.61)  26 (NA)  20 (1.41)  24.98 (2.90)  25.50 (2.68) |
| **WHrs/dy**  0=5  4=1  6=5  7=18  8=148  8.5=2  9=40  10=6  12=1  24=4 | **0.253**  24.40 (2.61)  27 (NA)  24.40 (2.70)  23.83 (2.97)  24.96 (2.98)  25 (2.83)  25.42 (2.64)  24.33 (3.50)  27 (NA)  26 (0.82) |
| **YrsExp1**  [0-10] =157  [11-20] =61  [21-30] =12 | **0.142**  24.72 (3.01)  25.57 (2.44)  (3.08) |
| **Work2**  NoOtherFieldofWrk=192  OtherFieldPh=29  OtherField=9 | **0.886**  24.91 (2.89)  25.17 (3.07)  25.11 (2.52) |
| **CompUnderGrad1**  [0-25] =107  [26-50] =79  [51-75] =29  [76-100] =15 | **0.760**  25.13 (2.80)  24.89 (2.52)  24.72 (3.06)  24.40 (4.7) |
| **CompPostGrad1**  [0-25] =109  [26-50] =46  [51-75] =31  [76-100] =44 | **0.563**  25.04 (2.57)  24.78 (3.33)  24.39 (3.01)  25.29 (3.09) |
| **CompCE1**  [0-25] =126  [26-50] =50  [51-75] =20  [76-100] =34 | **0.538**  24.99 (2.85)  25.10 (2.37)  24.05 (4.26)  25.09 (2.78) |
| **CompExp1**  [0-25] =11  [26-50] =65  [51-75] =46  [76-100] =108 | **0.359**  26.18 (1.66)  24.58 (3.03)  24.89 (2.81)  25.06 (2.92) |

**Table S5:** Bivariate Analysis *– Correlates of Professional Communication Skills*

| **Professional Communication Skills** | | | | | | | | | | | |
| --- | --- | --- | --- | --- | --- | --- | --- | --- | --- | --- | --- |
| **Variable** | **p-value**  **Mean (SD)** | | | | | | | | | | |
|  | PCSC | PCSN | PCSD | PCSI | PCSSMS | PCSMS | PCSP | PCSE | PCSL | PCSR | PCST |
| **Gender**  Male=65  Female=165 | **0.747**  38.83 (3.77)  39.01  (3.69) | **0.692**  32.28  (2.30)  32.10  (3.35) | **0.745**  23.69  (2.63)  23.54  (3.23) | **0.522**  13.66  (1.83)  13.83  (1.78) | **0.672**  27.78  (2.94)  28.00 (3.53) | **0.960**  33.94  (3.46)  33.90  (4.19) | **0.549**  20.69  (2.10)  20.89  (2.32) | **0.220**  11.85  (1.71)  12.13  (1.50) | **0.080**  26.84  (4.14)  27.74  (3.16) | **0.035***  13.65  (2.04)  14.14  (1.41) | **0.176**  7.49  (0.94)  7.66  (0.81) |
| **BS**  Yes=196  No=34 | **0.979**  38.96  (3.72)  38.94  (3.67) | **0.811**  32.13  (3.08)  32.26  (3.16) | **0.548**  23.64  (3.07)  23.29  (3.12) | **0.634**  13.80  (1.75)  13.65  (2.04) | **0.385**  28.01  (3.27)  27.47 (3.92) | **0.540**  33.98  (3.64)  33.53  (5.63) | **0.206**  20.91  (2.19)  20.38  (2.59) | **0.207**  12.10  (1.53)  11.73  (1.69) | **0.611**  27.53  (3.48)  27.20  (3.50) | **0.745**  13.99  (1.65)  14.09  (1.46) | **0.490**  7.60  (0.88)  7.70  (0.63) |
| **PharmD/DPharm**  Yes=92  No=138 | **0.913**  38.92  (4.20)  38.98  (3.35) | **0.326**  31.90  (3.78)  32.31  (2.53) | **0.255**  23.30  (3.18)  23.77  (2.99) | **0.042***  13.49  (1.97)  13.98  (1.64) | **0.020***  27.30  (3.98)  28.35  (2.83) | **0.038**  33.25  (4.75)  34.36  (3.33) | **0.156**  20.58  (2.65)  21.01  (1.94) | **0.595**  12.05  (1.51)  12.04  (1.60) | **0.945**  27.47  (3.24)  27.50  (3.64) | **0.345**  13.88  (1.80)  14.09  (1.50) | **0.949**  7.61  (0.80)  7.61  (0.88) |
| **Master**  Yes=72  No=158 | **0.336**  39.30 (2.99)  38.80  (3.99) | **0.571**  32.32 (2.50)  32.07  (3.33) | **0.880**  23.54  (3.02)  23.61  (3.10) | **0.791**  13.74 (1.92)  13.80 (1.74) | **0.407**  28.21  (2.93)  27.81 (3.55) | **0.595**  34.12  (4.22)  33.82  (3.45) | **0.493**  20.99  (2.01)  20.76  (2.36) | **0.687**  11.99  (1.60)  12.07  (1.55) | **0.716**  27.61  (3.14)  27.43  (3.63) | **0.307**  14.17  (1.34)  13.93  (1.73) | **0.390**  7.54  (1.02)  7.64  (0.76) |
| **PhD**  Yes=8  No=222 | **0.920**  -  - | **0.121**  -  - | **0.807**  -  - | **0.694**  -  - | **0.595**  -  - | **0.520**  -  - | **0.564**  -  - | **0.601**  -  - | **0.917**  -  - | **0.313**  -  - | **0.991**  -  - |
| **HighDeg**  BS=94  PharmD/DPharm=34  Master=52  PhD or equivalent=6  Others=4 | **0.918**  39.04  (3.27)  38.77  (4.58)  38.92  (3.40)  39.17  (1.60)  40.50 (1) | **0.759**  32.24  (2.60)  31.86  (4.00)  32.21  (2.61)  32.50  (1.97)  33.75  (0.5) | **0.789**  23.78  (2.95)  23.36  (3.23)  23.40  (3.24)  24.67  (1.63)  23.75  (2.63) | **0.670**  13.94 (1.59)  13.55 (1.94)  13.75 (2.04)  14.33 (0.82)  14.00  (1.15) | **0.153**  28.32 (2.87)  27.13 (4.17)  28.17 (3.01)  28.83 (2.04)  29.25 (0.96) | **0.581**  34.11  (3.50)  33.51  (4.86)  33.88  (3.67)  36.17  (1.33)  34.00  (3.56) | **0.263**  21.06  (1.83)  20.40  (2.78)  20.86  (2.24)  21.50  (0.84)  22.00  (0) | **0.745**  12.11  (1.55)  12.07  (1.55)  11.85  (1.71)  12.67  (0.52)  12.00  (1.15) | **0.780**  27.40  (3.87)  27.44  (3.20)  27.61  (3.32)  29.00  (1.26)  26.25  (4.5) | **0.650**  14.05  (1.58)  13.86  (1.84)  13.98  (1.50)  14.67  (0.52)  14.75  (0.5) | **0.946**  7.62  (0.80)  7.66  (0.71)  7.54  (1.11)  7.67  (0.52)  7.50  (1) |
| **YearGrad1**  1989-1999=14  2000-2010=63  2011-2020=153 | **0.001***  40.28 (1.07)  40.17 (2.02)  38.33 (4.21) | **0.019***  33.36  (1.15)  32.84  (2.38)  31.75  (3.38) | **0.083**  23.36  (2.95)  24.19  (2.52)  23.27  (3.25) | **0.225**  13.78 (1.93)  14.11 (1.45)  13.65 (1.90) | **0.011***  28.78  (2.78)  28.89  (2.53)  27.46  (3.63) | **0.017***  34.57  (3.23)  35.05  (2.84)  33.40  (4.35) | **0.104**  20.64  (3.34)  21.35  (1.76)  20.64  (2.30) | **0.183**  12.43  (1.16)  12.28  (1.35)  11.91  (1.66) | **0.115**  29.07  (1.49)  27.79  (3.24)  27.21  (3.66) | **0.312**  13.86  (2.18)  14.27  (1.28)  13.91  (1.69) | **0.798**  7.14  (0.61)  7.65  (0.81)  7.59  (0.88) |
| **UniPh**  Lebanese American University=46  Lebanese International University=46  Saint Joseph University=46  Beirut Arab University=39  Lebanese University=32  Foreign University=18 | **0.262**  39.76 (2.31)  37.85 (3.51)  39.00 (5.19)  38.75 (3.34)  38.87  (3.34)  39.39  (3.96) | **0.151**  32.63  (2.25)  32.34  (2.62)  31.65  (4.33)  32.59  (2.73)  32.09  (3.26)  33.17  (2.25) | **0.498**  24.09  (2.55)  23.00  (2.97)  23.37  (3.06)  23.56  (3.72)  23.44  (3.12)  24.39  (3.11) | **0.470**  14.15  (1.35)  13.63  (1.52)  13.52  (2.22)  13.59 (1.91)  14.06  (1.37)  13.61 (2.57) | **0.127**  28.67  (2.44)  26.93 (3.52)  27.72 (3.95)  28.08  (3.25)  27.75 (3.78)  29.05  (2.60) | **0.096**  35.22  (2.69)  33.00  (3.46)  33.83  (3.96)  33.36  (5.38)  33.53  (4.45)  34.83  (3.33) | **0.279**  21.17  (1.61)  20.61  (2.11)  20.65  (2.56)  21.28  (1.67)  20.25  (3.18)  21.28  (1.93) | **0.065**  12.46  (1.17)  11.54  (1.87)  12.09  (1.56)  12.28  (1.37)  11.81  (1.67)  12.33  (1.37) | **0.413**  27.85  (3.32)  26.56  (4.06)  27.78  (3.28)  27.74  (3.53)  27.37  (3.10)  28.17  (2.87) | **0.091**  14.19  (1.31)  13.63  (1.66)  14.28  (1.22)  14.13  (1.76)  13.47  (2.31)  14.44  (1.04) | **0.337**  7.85  (0.51)  7.48  (0.86)  7.65  (0.67)  7.49  (7.49)  7.56  (0.98)  7.61  (1.04) |
| **UniHighDeg**  Lebanese American University=38  Lebanese International University=37  Saint Joseph University=45  Beirut Arab University=30  Lebanese University=40  OtherLeb=19  OtherForeign=19 | **0.476**  39.60  (2.50)  38.08  (3.48)  39.00  (5.27)  38.57  (3.66)  38.67  (3.31)  39.84  (2.83)  39.64 (3.43) | **0.167**  32.44  (2.52)  31.46  (2.57)  31.64  (4.41)  32.33  (3.00)  31.82  (3.36)  33.21  (1.36)  33.37  (1.30) | **0.415**  24.08  (2.63)  23.04  (3.10)  23.47  (3.05)  23.90  (3.39)  23.00  (3.53)  24.31  (2.29)  24.31  (2.81) | **0.670**  14.03  (1.44)  13.54 (1.57)  13.78 (2.04)  13.40 (2.02)  14.07 (1.46)  14.00 (1.85)  13.74  (2.40) | **0.182**  28.63  (2.61)  26.86  (3.49)  28.15  (3.72)  27.80  (3.59)  27.52  (3.95)  27.95  (2.88)  29.21  (1.93) | **0.072**  35.10  (2.82)  32.84  (3.41)  34.18  (3.85)  32.90  (5.84)  33.45  (4.41)  35.21  (2.42)  34.68  (3.38) | **0.219**  21.05  (1.72)  20.57  (2.07)  20.75  (2.49)  21.27  (1.78)  20.17  (3.17)  21.42  (1.64)  21.47  (1.50) | **0.385**  12.34  (1.26)  11.70  (1.84)  12.09  (1.61)  12.23  (1.41)  11.77  (1.73)  12.26  (1.28)  12.42  (1.22) | **0.391**  27.47  (3.53)  26.67  (3.91)  27.71  (3.39)  27.63  (3.57)  27.32  (2.93)  27.68  (4.18)  29.05  (2.20) | **0.144**  14.03  (1.38)  13.73  (1.63)  14.24  (1.25)  14.17  (1.84)  13.52  (2.22)  14.21  (1.44)  14.68  (0.58) | **0.065**  7.87  (0.47)  7.43  (0.90)  7.69  (0.67)  7.69  (0.86)  7.35  (1.29)  7.68  (0.67)  7.89  (0.31) |
| **Lang**  English=146  French=78  Other=6 | **0.571**  38.96  (3.24)  38.83  (4.56)  40.50  (0.84) | **0.193**  32.35  (2.50)  31.68  (4.00)  33.33  (1.63) | **0.357**  24.64  (3.12)  23.36  (3.05)  25.17  (1.33) | **0.739**  13.85  (1.67)  13.68  (1.97)  13.50 (2.51) | **0.241**  28.00 (3.08)  27.65  (3.94)  30.00  (0) | **0.365**  33.94 (3.95)  33.73 (4.17)  35.67 (2.06) | **0.272**  20.94  (2.08)  20.56  (2.60)  21.83  (0.41) | **0.500**  12.12  (1.53)  11.88  (1.64)  12.33  (1.03) | **0.609**  27.40  (3.66)  27.54  (3.24)  28.83  (1.47) | **0.400**  14.02  (1.54)  13.91  (1.82)  14.83  (0.41) | **0.385**  7.64  (0.82)  7.59  (0.83)  7.17  (1.60) |
| **WLoc**  Beirut=121  Beqaa=12  Mount Lebanon=44  North Lebanon=29  South Lebanon=8  Currently not working=16 | **0.902**  38.83 (4.33)  38.67  (2.31)  39.32  (2.72)  38.55 (3.71)  39.75 (1.58)  39.44 (2.42) | **0.806**  31.93  (3.57)  32.50  (1.93)  32.64  (1.99)  31.90  (3.31)  32.25  (1.83)  32.56  (2.41) | **0.677**  23.34  (3.21)  23.50  (2.71)  23.52  (3.40)  24.13  (2.67)  24.37  (2.13)  24.31  (2.30) | **0.050**  13.47 (2.10)  13.83  (1.53)  14.50  (1)  13.83  (1.58)  13.87 (0.83)  14.00 (1.41) | **0.415**  27.63  (3.77)  27.08  (3.75)  28.79  (3.20)  27.96  (3.31)  28.62  (1.85)  28.12  (3.20) | **0.365**  33.49  (4.49)  33.42  (3.34)  35.09  (2.66)  34.14  (3.50)  34.00  (3.58)  33.81  (4.31) | **0.903**  20.69  (2.38)  21.00  (1.35)  21.09  (2.21)  20.72  (2.50)  21.00  (1.60)  21.19  (1.83) | **0.443**  12.05  (1.61)  12.17  (1.11)  12.39  (1.33)  11.86  (1.73)  11.75  (1.16)  11.50  (1.90) | **0.238**  27.35  (3.75)  27.75  (2.05)  28.18  (3.07)  27.48  (3.15)  27.75  (2.12)  26.25  (4.30) | **0.238**  13.94  (1.72)  13.17  (2.29)  14.39  (1.20)  13.83  (1.60)  14.12  (1.35)  14.31  (1.25) | **0.994**  7.62  (0.89)  7.58  (0.67)  7.66  (0.80)  7.55  (0.87)  7.50  (0.75)  7.62  (0.81) |
| **Wdays/wk**  0=5  2=1  3=2  5=214  6=8 | **0.479**  38.00 (3.67)  -  (NA)  34.50  (6.36)  39.08  (3.60)  37.37  (5.58) | **0.107**  30.40  (3.78)  34.00  (NA)  26.50  (9.19)  32.23  (2.99)  32.12  (2.69) | **0.126**  21.80  (4.76)  26.00  (NA)  18.00  (1.410  23.68  (2.99)  23.25  (3.33) | **0.470**  13.40  (1.81)  15.00  (NA)  11.00 (0)  13.82 (1.79)  13.62 (1.68) | **0.005**  24.60  (4.98)  27.00  (NA)  19.00  (2.83)  28.14 (3.16)  26.75  (4.37) | **0.564**  33.20  (3.90)  37.00  (NA)  27.00  (5.66)  34.02  (3.90)  32.87  (5.17) | **0.086**  19.60  (2.61)  21.00  (NA)  15.00  (2.83)  20.95  (2.14)  20.00  (3.16) | **0.266**  11.40  (2.19)  13.00  (NA)  9.00  (1.41)  12.11  (1.51)  11.50  (1.85) | **0.856**  27.20  (3.11)  28.00  (NA)  24.00  (4.24)  27.58  (3.47)  26.00  (3.93) | **0.445**  13.00  (1.87)  15.00  (NA)  11.50  (2.12)  14.13  (1.40)  11.87  (3.91) | **0.455**  7.40  (0.89)  7.00  (NA)  6.50  (0.71)  7.65  (0.82)  7.12  (1.25) |
| **WHrs/dy**  0=5  4=1  6=5  7=18  8=148  8.5=2  9=40  10=6  12=1  24=4 | **0.704**  38.00  (3.67)  41.00  (NA)  40.00  (1.41)  38.05  (4.23)  38.96  (3.29)  40.00  (1.41)  39.25  (5.19)  38.50  (3.99)  41.00  (NA)  39.00  (1.82) | **0.868**  30.40  (3.78)  34.00  (NA)  33.20  (0.84)  31.00  (3.76)  32.22  (2.62)  32.00  (2.83)  32.58  (4.22)  31.83  (3.71)  34.00  (NA)  31.00  (3.56) | **0.483**  21.80  (4.76)  26.00  (NA)  24.00  (3.08)  22.78  (2.96)  23.50  (2.99)  19.50  (9.19)  24.52  (2.74)  24.00  (3.16)  26.00  (NA)  23.00  (3.16) | **0.058**  13.40 (1.82)  15.00 (NA)  14.00  (1.73)  13.78  (1.39)  13.74  (1.80)  15.00  (0)  14.15  (1.79)  13.67  (2.06)  12.00  (NA)  11.50  (2.64) | **0.996**  24.60  (4.98)  30.00  (NA)  27.40  (3.78)  27.78  (3.64)  27.90  (3.27)  28.50  (2.12)  28.78  (3.25)  28.00  3.63)  29.00  (NA)  25.25  (3.77) | **0.627**  33.20  (3.90)  37.00  (NA)  32.40  (3.71)  32.67  (4.58)  33.88  (4.07)  36.50  (0.71)  34.72  (3.51)  35.17  (3.60)  36.00  (NA)  31.00  (4.08) | **0.102**  19.60  (2.61)  22.00  (NA)  19.80  (2.17)  20.50  (2.81)  20.76  (2.28)  22.00  (0)  21.37  (1.99)  20.67  (2.42)  21.00  (0)  21.75  (0.5) | **0.331**  11.4  (2.19)  13.00  (NA)  12.00  (1.41)  11.05  (2.07)  12.10  (1.51)  13.00  (0)  12.60  (0.98)  11.17  (2.04)  13.00  (NA)  10.25  (1.90) | **0.998**  27.20  (3.11)  30.00  (NA)  27.80  (1.920  26.33  (3.56)  27.50  (3.59)  29.50  (0.710  27.95  (3.35)  26.50  (4.04)  28.00  (NA)  27.00  (3.56) | **0.260**  13.00  (1.87)  15.00  (NA)  14.60  (0.89)  13.39  (2.57)  14.03  (1.49)  15.00  (0)  14.35  (1.14)  13.83  (1.60)  15.00  (NA)  12.00  (3.83) | **0.008***  7.40  (0.89)  8.00  (NA)  7.60  (0.55)  7.44  (0.98)  7.63  (0.79)  8.00  (0)  7.80  (0.85)  7.50  (0.84)  8.00  (NA)  6.00  (1.41) |
| **YrsExp1**  [0-10] =157  [11-20] =61  [21-30] =12 | **0.001***  38.35  (4.15)  40.23  (2.12)  40.42  (0.90) | **0.006***  33.71  (3.42)  33.05  (2.04)  33.33  (1.23) | **0.005***  23.14  (3.25)  24.52  (2.33)  24.67  (2.71) | **0.228**  13.65  (1.88)  14.11 (1.49)  13.83 (1.99) | **0.006***  27.46  (3.65)  28.90  (2.35)  29.25  (2.60) | **0.003***  33.32  (4.33)  35.18  (2.79)  35.33  (2.57) | **0.137**  20.64  (2.33)  21.21  (2.13)  21.50  (1.44) | **0.050***  11.88  (1.66)  12.38  (1.29)  12.58  (1.00) | **0.030***  27.11  (3.69)  28.08  (3.03)  29.33  (0.98) | **0.134**  13.87  (1.75)  14.24  (1.35)  14.58  (0.67) | **0.486**  7.57  (0.89)  7.67  (0.79)  7.83  (0.39) |
| **Work2**  NoOtherFieldofWrk=192  OtherFieldPh=29  OtherField=9 | **0.194**  39.15  (3.07)  38.07  (6.39)  37.67  (4.33) | **0.061**  32.36  (2.57)  31.17  (5.29)  30.78  (3.31) | **0.111**  23.76  (2.91)  22.48  (3.92)  23.44  (2.83) | **0.029***  13.92 (1.54)  13.07 (2.74)  13.11 (2.57) | **0.023***  28.20 (2.95)  26.79  (4.99)  26.00  (4.30) | **0.040***  34.18  (3.55)  32.17 (6.06)  33.89 (3.55) | **0.640**  20.89  (2.11)  20.48  (2.98)  20.67  (2.64) | **0.367**  12.07  (1.54)  12.14  (1.48)  11.33  (2.12) | **0.997**  27.48  (3.50)  27.51  (3.59)  27.56  (3.09) | **0.838**  13.98  (1.65)  14.17  (1.46)  14.00  (1.73) | **0.533**  7.63  (0.79)  7.55  (1.15)  7.33  (1) |
| **CompUnderGrad1**  [0-25] =107  [26-50] =79  [51-75] =29  [76-100] =15 | **<0.001***  39.77  (2.30) 38.66  (3.23)  36.62  (6.89)  39.27  (3.79) | **0.132**  32.37  (2.72)  31.95  (2.66)  31.24  (5.21)  33.33  (1.59) | **0.085**  23.77  (2.83)  23.32  (3.16)  22.83  (3.97)  25.13  (1.35) | **0.497**  13.86  (1.57)  13.63  (2.08)  13.62  (2.04)  14.33  (0.97) | **0.211**  28.21  (3.06)  27.69  (3.24)  27.03  (4.85)  28.93  (2.37) | **0.140**  34.10  (3.72)  33.64  (3.32)  33.00  (6.44)  35.80  (2.24) | **0.077**  21.08  (1.88)  20.71  (2.32)  19.96  (3.27)  21.40  (1.55) | **0.766**  12.13  (1.42)  12.05  (1.55)  11.79  (1.78)  11.93  (2.15) | **0.704**  27.25  (3.64)  27.75  (2.85)  27.31  (3.49)  28.00  (5.17) | **0.739**  14.05  (1.63)  14.06  (1.31)  13.68  (2.27)  14.00  (1.65) | **0.290**  7.73  (0.72)  7.51  (0.97)  7.52  (0.87)  7.53  (0.91) |
| **CompPostGrad1**  [0-25] =109  [26-50] =46  [51-75] =31  [76-100] =44 | **<0.001***  39.54  (2.59)  39.33  (2.32) 36.29  (6.25)  39.00  (4.15) | **0.010***  32.27  (2.75)  32.39  (2.32)  30.48  (5.12)  32.75  (2.33) | **0.111**  23.87  (2.78)  23.17  (3.22)  22.58  (3.78)  24.02  (2.94) | **0.217**  13.83  (1.76)  13.91  (1.38)  13.16  (2.31)  13.95  (1.82) | **0.008***  28.15  (3.10)  28.23  (2.26)  26.03  (4.86)  28.43  (3.38) | **0.010***  34.12  (3.62)  33.93  (3.37)  31.84  (5.94)  34.86  (3.33) | **<0.001***  21.07  (1.91)  21.26  (1.36)  19.22  (3.64)  20.93  (2.13) | **0.561**  12.08  (1.51)  12.13  (1.42)  11.68  (1.68)  12.13  (1.75) | **0.570**  27.23  (3.59)  27.50  (3.01)  27.48  (3.50)  28.11  (3.69) | **0.807**  14.04  (1.63)  14.13  (1.24)  13.93  (1.44)  13.82  (2.05) | **0.744**  7.60  (0.91)  7.61  (0.85)  7.52  (0.72)  7.72  (0.76) |
| **CompCE1**  [0-25] =126  [26-50] =50  [51-75] =20  [76-100] =34 | **0.002***  39.29  (2.84)  39.48  (2.64)  36.05  (5.17)  38.65  (5.74) | **0.639**  32.67  (2.67)  32.04  (2.79)  31.35  (3.17)  32.32  (4.63) | **0.076**  23.87  (2.70)  23.14  (3.20)  22.20  (3.87)  24.00  (3.45) | **0.453**  13.81  (1.80)  13.80  (1.60)  13.20  (2.17)  14.00  (1.81) | **0.149**  28.25  (2.86)  27.54  (3.66)  26.55  (4.06)  28.15  (4.08) | **0.182**  34.02  (3.62)  34.02  (3.48)  32.10  (4.01)  34.44  (5.58) | **0.002***  21.05  (1.95)  20.84  (1.96)  19.05  (3.46)  21.09  (2.45) | **0.024***  12.09  (1.52)  12.06  (1.43)  11.10  (2.27)  12.41  (1.21) | **0.052**  27.40  (3.33)  27.36  (3.35)  26.15  (4.94)  28.76  (2.89) | **0.016***  14.09  (1.55)  14.16  (1.33)  12.90  (2.59)  14.11  (1.34) | **0.257**  7.55  (0.92)  7.74  (0.72)  7.40  (1.05)  7.76  (0.550 |
| **CompExp1**  [0-25] =11  [26-50] =65  [51-75] =46  [76-100] =108 | **0.403**  39.27  (2.72)  39.48  (2.82)  39.15  (3.15)  38.53  (4.40) | **0.989**  32.45  (2.77)  32.15  (2.82)  32.15  (2.61)  32.11  (3.47) | **0.840**  24.00  (2.45)  23.75  (2.66)  23.28  (3.34)  23.57  (3.25) | **0.892**  14.00  (1.34)  13.66  (1.77)  13.89  (1.82)  13.79  (1.85) | **0.989**  28.00  (3.19)  28.05  (3.16)  27.93  (3.40)  27.86  (3.53) | **0.855**  34.64  (3.77)  34.08  (3.33)  33.59  (3.67)  33.89  (4.51) | **0.738**  21.18  (1.66)  20.77  (2.13)  20.56  (2.78)  20.95  (2.14) | **0.885**  12.09  (1.58)  11.92  (1.58)  12.04  (1.52)  12.12  (1.58) | **0.330**  28.18  (2.44)  27.26  (3.10)  26.82  (3.63)  27.83  (3.70) | **0.931**  13.72  (2.53)  14.01  (1.52)  14.09  (1.50)  13.99  (1.64) | **0.771**  7.72  (0.65)  7.52  (1.03)  7.63  (0.85)  7.65  (0.74) |

**Table S6:** Bivariate Analysis *– Correlates of Pharmacists’ Preparedness and Response in*

*Emergency Situations*

| **Pharmacist Preparedness and Response in Emergency Situations** | | | | |
| --- | --- | --- | --- | --- |
| **Variable** | **p-value**  **Mean (SD)** | | | |
|  | **PPE** | **PPO** | **PPC** | **PPERD** |
| **Gender**  Male=65  Female=165 | **0.312**  30.00(5.34)  30.68(4.29) | **0.666**  34.38(5.94)  34.76(6.02) | **0.780**  7.94(1.80)  7.87(1.73) | **0.652**  15.03(3.88)  15.27(3.57) |
| **BS**  Yes=196  No=34 | **0.119**  30.69(4.33)  20.35(5.91) | **0.571**  34.75(6.07)  34.12(5.54) | **0.388**  7.93(1.74)  7.65(1.82) | **0.479**  15.27(3.56)  14.79(4.19) |
| **PharmD/DPharm**  Yes=92  No=138 | **0.395**  30.17(4.62)  30.70(4.61) | **0.017***  33.51(6.82)  35.42(5.25) | **0.058**  7.62(1.80)  8.06(1.70) | **0.153**  14.78(3.84)  15.48(3.51) |
| **Master**  Yes=72  No=158 | **0.773**  30.36(5.00)  30.55(4.44) | **0.078**  33.62(6.84)  35.12(5.51) | **0.380**  7.74(2.17)  7.95(1.52) | **0.516**  14.97(4.51)  15.31(3.20) |
| **PhD**  Yes=8  No=222 | **0.773**  -  - | **0.586**  -  - | **0.889**  -  - | **0.639**  -  - |
| **HighDeg**  BS=94  PharmD/DPharm=34  Master=52  PhD or equivalent=6  Others=4 | **0.241**  30.72(4.40)  30.28(4.39)  30.11(5.36)  34.17(1.60)  28.25(5.00) | **0.055**  35.39(5.51)  34.47(5.69)  33.38(7.06)  39.17(0.98)  30.50(6.80) | **0.048***  8.01(1.60)  7.88(1.48)  7.60(2.22)  9.50(0.84)  6.50(2.52) | **0.021**  15.42(3.24)  15.17(3.12)  14.38(4.79)  19.50(0.84)  14.75(3.77) |
| **YearGrad1**  1989-1999=14  2000-2010=63  2011-2020=153 | **0.236**  32.28(4.25)  29.98(5.10)  30.53(4.41) | **0.050***  38.43(2.82)  34.30(6.15)  34.46(6.04) | **0.127**  8.78(1.53)  7.75(2.05)  7.86(1.62) | **0.815**  15.78(4.24)  15.09(3.59)  15.20(3.64) |
| **UniPh**  Lebanese American University=46  Lebanese International University=46  Saint Joseph University of Beirut=46  Beirut Arab University=39  Lebanese University=32  Foreign University=18 | **0.085**  32.13(3.34)  29.54(3.96)  30.30(4.15)  30.15(4.86)  30.03(5.86)  31.55(5.38) | **0.025***  36.28(5.34)  33.74(5.65)  32.85(7.52)  35.15(5.36)  34.65(5.39)  37.28(3.82) | **0.048***  7.96(1.71)  7.98(1.39)  7.41(1.79)  7.72(1.83)  8.16(1.78)  8.87(1.45) | **0.199**  15.69(3.41)  14.50(3.07)  14.78(3.68)  15.23(3.64)  15.41(4.27)  16.87(3.41) |
| **UniHighDeg**  Lebanese American University=38  Lebanese International University=37  Saint Joseph University of Beirut=45  Beirut Arab University=30  Lebanese University=40  OtherLeb=19  OtherForeign=19 | **0.426**  31.92(3.07)  29.92(3.99)  30.58(3.87)  29.97(5.13)  30.30(5.31)  29.68(5.92)  31.37(5.56) | **0.174**  36.39(4.40)  34.22(5.99)  34.11(6.22)  35.43(5.23)  34.27(5.26)  32.05(8.83)  36.00(6.84) | **0.232**  7.87(1.63)  8.16(1.30)  7.78(1.50)  7.70(1.86)  8.10(1.69)  7.05(2.86)  8.42(1.74) | **0.289**  15.55(2.88)  14.89(3.23)  15.40(3.07)  15.60(3.21)  15.27(3.93)  13.31(5.64)  16.10(4.37) |
| **Lang**  English=146  French=78  Other=6 | **0.287**  30.64(4.50)  30.02(4.87)  32.83(3.12) | **0.021***  35.27(5.460  33.25(6.77)  38.00(4) | **0.218**  8.00(1.71)  7.63(1.79)  8.50(1.97) | **0.639**  15.23(3.49)  15.05(3.85)  16.50(5.20) |
| **WLoc**  Beirut=121  Beqaa=12  Mount Lebanon=44  North Lebanon=29  South Lebanon=8  Currently not working=16 | **0.537**  30.11(4.88)  31.50(4.76)  31.52(3.60)  29.96(5.08)  31.12(3.83)  30.37(4.42) | **0.067**  33.70(6.53)  35.33(7.24)  36.82(4.25)  34.10(5.63)  35.37(5.47)  36.06(4.17) | **0.696**  7.73(1.81)  8.00(1.70)  8.04(1.40)  8.24(1.80)  8.25(1.67)  7.75(2.21) | **0.997**  15.24(3.52)  15.17(5.29)  15.29(3.61)  14.86(3.92)  15.50(3.21)  15.19(3.60) |
| **Wdays/wk**  0=5  2=1  3=2  5=214  6=8 | **0.932**  30.60(5.13)  33.00(NA)  26.50(2.12)  30.52(4.47)  30.37(8.22) | **0.997**  35.60(4.56)  38.00(NA)  31.50(0.71)  34.55(6.09)  37.25(4.37) | **0.877**  8.00(1.41)  8.00(NA)  7.50(0.71)  7.86(1.77)  8.50(1.69) | **0.687**  15.60(2.07)  16.00(NA)  10.00(2.83)  15.20(3.67)  16.37(3.78) |
| **WHrs/dy**  0=5  4=1  6=5  7=18  8=148  8.5=2  9=40  10=6  12=1  24=4 | **0.576**  30.60(5.13)  35.00(NA)  31.00(3.08)  26.83(5.65)  30.67(4.58)  32.00(4.24)  31.60(3.77)  29.67(3.26)  31.00(NA)  27.75(6.29) | **0.504**  35.60(4.56)  40.00(NA)  36.40(4.10)  30.44(6.37)  35.04(5.64)  39.50(0.71)  35.22(6.65)  31.33(6.310  30.00(NA)  32.75(8.46) | **0.818**  8.00(1.41)  10.00(NA)  8.00(0)  7.33(1.53)  7.85(1.80)  9.00(1.41)  8.15(1.85)  8.00(1.79)  8.00(NA)  7.50(1.94) | **0.999**  15.60(2.07)  16.00(NA)  13.60(3.29)  14.39(2.40)  15.26(3.82)  18.00(2.83)  15.40(3.92)  15.50(3.08)  12.00(NA)  15.00(3.46) |
| **YrsExp1**  [0-10] =157  [11-20] =61  [21-30] =12 | **0.434**  30.41(4.45)  30.36(5.03)  32.17(4.53) | **0.099**  34.52(5.97)  34.29(6.29)  38.25(3.02) | **0.214**  7.83(1.65)  7.85(2.02)  8.75(1.36) | **0.414**  14.99(3.70)  15.59(3.42)  16.00(4.20) |
| **Work2**  NoOtherFieldofWrk=192  OtherFieldPh=29  OtherField | **0.052**  30.31(4.71)  32.24(3.83)  28.67(3.57) | **0.032***  34.55(5.90)  36.59(4.41)  30.78(9.82) | **0.289**  7.83(1.76)  8.34(1.39)  7.55(2.45) | **0.297**  15.17(3.55)  15.90(3.97)  13.78(4.66) |
| **CompUnderGrad1**  [0-25] =107  [26-50] =79  [51-75] =29  [76-100] =15 | **0.006***  29.83(4.54)  30.49(4.57)  31.00(5.17)  34.20(1.90) | **<0.001***  33.12(6.63)  35.34(5.41)  36.72(3.62)  38.00(4.66) | **0.001***  7.52(1.92)  7.96(1.51)  8.31(1.42)  9.27(1.33) | **0.0013**  14.53(3.91)  15.23(3.44)  16.07(2.96)  18.20(2.11) |
| **CompPostGrad1**  [0-25] =109  [26-50] =46  [51-75] =31  [76-100] =44 | **0.084**  29.78(4.57)  30.43(4.19)  31.61(3.15)  31.52(5.67) | **0.008***  33.46(6.38)  34.61(6.41)  35.77(3.59)  36.88**(4.85)** | **0.005***  7.65(1.75)  7.54(1.87)  8.16(1.39)  8.63(1.64) | **<0.001***  14.87(3.68)  13.78(4.05)  15.64(2.75)  17.20(2.82) |
| **CompCE1**  [0-25] =126  [26-50] =50  [51-75] =20  [76-100] =34 | **0.022***  29.89(4.56)  31.20(3.95)  29.45(6.63)  32.29(3.74) | **0.022***  33.71(6.69)  35.16(5.26)  34.80(4.64)  37.32**(3.82)** | **0.006***  7.57(1.83)  8.06(1.57)  8.05(1.28)  8.70(1.70**)** | **0.006***  14.46(3.97)  15.96(3.05)  14.95(2.91)  17.00(2.77) |
| **CompExp1**  [0-25] =11  [26-50] =65  [51-75] =46  [76-100] =108 | **0.420**  31.91(3.24)  29.95(4.74)  30.11(4.15)  30.83(4.83) | **0.034***  37.64(4.27)  33.40(5.97)  33.78(6.39)  35.48(5.79) | **0.028***  8.72(1.35)  7.52(1.79)  7.56(1.57)  8.15(1.77) | **0.017***  15.36(4.34)  14.46(3.85)  14.37(3.29)  15.99(3.48) |

**Table S7:** Multivariable analysis of sales and marketing competencies

| **Variable** | **Estimate** | **Standard error** | **Lower CI** | **Upper CI** | **p-value** |
| --- | --- | --- | --- | --- | --- |
| **Pharmaceutical Knowledge** | | | | | |
| **HighDeg**  BS=94  PharmD/DPharm=34  Master=52  PhD or equivalent=6  Others=4 | -0.498  -0.157  0.389  -5.146 | 0.445  0.497  1.201  1.461 | -1.369  -1.136  -1.972  -7.995 | 0.381  0.816  2.751  -2.262 | 0.265  0.753  0.746  **<0.001*** |
| **YearGrad1**  1989-1999=14  2000-2010=63  2011-2020=153 | -0.055  -0.857 | 1.541  1.732 | -2.838  -3.699 | 2.909  2.291 | 0.971  0.621 |
| **YrsExp1**  [0-10] =157  [11-20] =61  [21-30] =12 | 0.306  -0.608 | 0.713  1.705 | -1.080  -3.912 | 1.621  2.514 | 0.668  0.721 |
| **Professional Communication Skills** | | | | | |
| **Communication** | | | | | |
| **YrsExp1**  [0-10] =157  [11-20] =61  [21-30] =12 | 1.190  0.250 | 0.871  1.965 | -0.526  -3.624 | 2.907  4.123 | 0.173  0.900 |
| **Work2**  OtherFieldPh=29  NoOtherFieldofWrk=192  OtherField=9 | 0.547  0.041 | 0.713  1.322 | -0.857  -2.566 | 1.952  2.648 | 0.443  0.975 |
| **CompUnderGrad1**  [0-25] =107  [26-50] =79  [51-75] =29  [76-100] =15 | -0.336  -2.566  -0.205 | 0.549  0.807  1.067 | -1.417  -4.158  -2.306 | 0.746  -0.975  1.897 | 0.541  **0.002***  0.848 |
| **CompPostGrad1**  [0-25] =109  [26-50] =46  [51-75] =31  [76-100] =44 | 0.331  -1.970  0.024 | 0.636  0.840  0.835 | -0.921  -3.625  -1.622 | 1.585  -0.315  1.670 | 0.603  **0.020***  0.977 |
| **CompCE1**  [0-25] =126  [26-50] =50  [51-75] =20  [76-100] =34 | 0.009  -2.172  -0.010 | 0.612  0.935  0.949 | -1.197  -4.015  -1.881 | 1.215  -0.328  1.861 | 0.988  **0.021***  0.991 |
| **YearGrad1**  1989-1999=14  2000-2010=63  2011-2020=153 | -1.408  -1.966 | 1.776  1.836 | -4.908  -5.585 | 2.092  1.654 | 0.429  0.286 |
| **Negotiation** | | | | | |
| **YearGrad1**  1989-1999=14  2000-2010=63  2011-2020=153 | -0.927  -1.613 | 1.554  1.613 | -3.991  -4.253 | 2.136  2.103 | 0.551  0.505 |
| **YrsExp1**  [0-10] =157  [11-20] =61  [21-30] =12 | 1.063  0.512 | 0.732  1.726 | -0.379  -2.890 | 2.136  2.103 | 0.148  0.767 |
| **CompPostGrad1**  [0-25] =109  [26-50] =46  [51-75] =31  [76-100] =44 | 0.282  -1.648  0.254 | 0.531  0.616  0.543 | -0.765  -2.862  -0.817 | 1.328  -0.433  1.324 | 0.597  **0.008***  0.641 |

| **Data Processing Analysis** | | | | | | |
| --- | --- | --- | --- | --- | --- | --- |
| **YrsExp1**  [0-10] =157  [11-20] =61  [21-30] =12 | | 1.860  2.231 | 0.762  1.721 | 0.359  -1.161 | 3.362  5.623 | **0.015***  0.196 |
| **Work2**  OtherFieldPh=29  NoOtherFieldofWrk=192  OtherField=9 | | 1.077  1.059 | 0.625  1.156 | -0.155  -1.220 | 2.310  3.337 | 0.086  0.361 |
| **Wdays/wk** | | 0.259 | 0.257 | -0.248 | 0.766 | 0.315 |
| **CompUnderGrad1**  [0-25] =107  [26-50] =79  [51-75] =29  [76-100] =15 | | -0.146  -0.756  1.505 | 0.480  0.708  0.936 | -1.093  -2.152  -0.339 | 0.800  0.639  3.350 | 0.760  0.287  0.109 |
| **CompPostGrad1**  [0-25] =109  [26-50] =46  [51-75] =31  [76-100] =44 | | -0.227  -0.257  0.178 | 0.555  0.738  0.731 | -1.322  -1.711  -1.262 | 0.868  1.198  1.619 | 0.684  0.728  0.808 |
| **CompCE1**  [0-25] =126  [26-50] =50  [51-75] =20  [76-100] =34 | | -1.030  -1.569  -0.457 | 0.535  0.821  0.833 | -2.085  -3.187  -2.099 | 0.024  0.049  1.185 | 0.055  0.057  0.584 |
| **YearGrad1**  1989-1999=14  2000-2010=63  2011-2020=153 | | 0.037  0.757 | 1.557  1.609 | -3.033  -2.414 | 3.106  3.929 | 0.981  0.638 |
| **Information Technology** | | | | | | |
| **Work2**  OtherFieldPh=29  NoOtherFieldofWrk=192  OtherField=9 | 0.686  -0.184 | 0.354  0.679 | -0.013  -1.522 | | 1.384  1.154 | 0.054  0.787 |
| **Whrs/dy** | -0.077 | 0.047 | -0.171 | | 0.016 | 0.104 |
| **WLoc**  Beirut=121  Beqaa=12  Mount Lebanon=44  North Lebanon=29  South Lebanon=8  Currently not working=16 | 0.196  0.891  0.175  0.240  0.292 | 0.541  0.310  0.366  0.642  0.482 | -0.871  0.280  -0.546  -1.026  -0.658 | | 1.264  1.503  0.897  1.507  1.242 | 0.717  **0.004***  0.633  0.708  0.546 |
| **PharmD/DPharm**  No=138  Yes=92 | -0.432 | 0.244 | -0.913 | | 0.049 | 0.078 |
| **Self-Management Skills** | | | | | | |
| **YrsExp1**  [0-10] =157  [11-20] =61  [21-30] =12 | | 0.500  1.657 | 0.795  1.852 |  |  | 0.530  0.372 |
| **Work2**  OtherFieldPh=29  NoOtherFieldofWrk=192  OtherField=9 | | 0.839  -0.680 | 0.839  1.243 | -0.464  -3.130 | 2.141  1.770 | 0.206  0.584 |
| **PharmD/DPharm**  No=138  Yes=92 | | -1.096 | 0.440 | -1.963 | -0.229 | **0.013*** |
| **CompPostGrad1**  [0-25] =109  [26-50] =46  [51-75] =31  [76-100] =44 | | 0.254  -1.771  0.034 | 0.571  0.665  0.586 | -0.872  -3.082  -1.121 | 1.381  -0.460  1.187 | 0.656  **0.008***  0.955 |
| **YearGrad1**  1989-1999=14  2000-2010=63  2011-2020=153 | | 0.927  0.057 | 1.667  1.732 | -2.358  -3.358 | 4.211  3.472 | 0.578  0.974 |
| **Management Skills** | | | | | | |
| **YrsExp1**  [0-10] =157  [11-20] =61  [21-30] =12 | | 1.156  3.301 | 0.947  2.212 | -0.710  -1.059 | 3.022  7.662 | 0.223  0.137 |
| **Work2**  OtherFieldPh=29  NoOtherFieldofWrk=192  OtherField=9 | | 1.582  2.200 | 0.787  1.483 | 0.032  -0.722 | 3.132  5.123 | **0.045***  0.139 |
| **CompPostGrad1**  [0-25] =109  [26-50] =46  [51-75] =31  [76-100] =44 | | -0.070  -1.922  0.639 | 0.683  0.795  0.699 | -1.416  -3.489  -0.738 | 1.276  -0.355  2.017 | 0.918  **0.016***  0.361 |
| **YearGrad1**  1989-1999=14  2000-2010=63  2011-2020=153 | | 2.009  1.614 | 1.992  2.068 | -1.917  -2.461 | 5.937  5.689 | 0.314  0.436 |
| **Standard Practice** | | | | | | |
| **YrsExp1**  [0-10] =157  [11-20] =61  [21-30] =12 | | 0.290  2.825 | 0.535  1.256 | -0.766  0.350 | 1.345  5.300 | 0.589  **0.025*** |
| **PharmD/DPharm**  No=138  Yes=92 | | -0.515 | 0.300 | -1.107 | 0.078 | 0.088 |
| **CompUnderGrad1**  [0-25] =107  [26-50] =79  [51-75] =29  [76-100] =15 | | -0.074  -0.862  0.195 | 0.346  0.502  0.668 | -0.756  -1.852  -1.122 | 0.607  0.128  1.511 | 0.830  0.088  0.771 |
| **CompPostGrad1**  [0-25] =109  [26-50] =46  [51-75] =31  [76-100] =44 | | 0.540  -1.353  -0.202 | 0.395  0.519  0.523 | -0.239  -2.377  -1.233 | 1.320  -0.330  0.828 | 0.173  **0.010***  0.699 |
| **CompCE1**  [0-25] =126  [26-50] =50  [51-75] =20  [76-100] =34 | | -0.254  -1.203  0.594 | 0.378  0.574  0.590 | -1.000  -2.335  -0.569 | 0.491  -0.070  1.758 | 0.502  0.037  0.315 |
| **YearGrad1**  1989-1999=14  2000-2010=63  2011-2020=153 | | 2.601  2.235 | 1.139  1.180 | 0.355  -0.091 | 4.847  4.561 | **0.023***  0.059 |
| **Wdays/wk** | | -0.056 | 0.210 | -0.470 | 0.359 | 0.791 |
| **Whrs/dy** | | 0.096 | 0.067 | -0.036 | 0.227 | 0.153 |
| **Ethical Practice** | | | | | | |
| **YrsExp1**  [0-10] =157  [11-20] =61  [21-30] =12 | | 0.659  0.607 | 0.390  0.888 | -0.109  -1.143 | 1.428  2.357 | 0.092  0.495 |
| **CompCE1**  [0-25] =126  [26-50] =50  [51-75] =20  [76-100] =34 | | -0.159  -0.950  0.096 | 0.260  0.373  0.308 | -0.671  -1.686  -0.512 | 0.354  -0.214  0.703 | 0.542  **0.012***  0.756 |
| **YearGrad1**  1989-1999=14  2000-2010=63  2011-2020=153 | | -0.072  0.050 | 0.794  0.839 | -1.636  -1.604 | 1.492  1.705 | 0.928  0.952 |
| **UniPh**  Foreign University=18  LAU= 46  LIU=46  Saint Joseph University=46  Beirut Arab University=39  Lebanese University=32 | | 0.243  -0.582  -0.185  0.014  -0.477 | 0.444  0.442  0.436  0.442  0.454 | -0.633  -1.453  -1.045  -0.858  -1.371 | 1.119  0.288  0.675  0.886  1.119 | 0.584  0.189  0.672  0.974  0.294 |
| **Legal Practice** | | | | | | |
| **YrsExp1**  [0-10] =157  [11-20] =61  [21-30] =12 | | 1.221  1.863 | 0.850  1.971 | -0.454  -2.021 | 2.895  5.748 | 0.152  0.345 |
| **CompCE1**  [0-25] =126  [26-50] =50  [51-75] =20  [76-100] =34 | | -0.185  -1.292  1.059 | 0.582  0.829  0.681 | -1.332  -2.926  -0.283 | 0.962  0.341  2.401 | 0.751  0.120  0.121 |
| **YearGrad1**  1989-1999=14  2000-2010=63  2011-2020=153 | | -0.600  0.012 | 1.780  1.846 | -4.107  -3.626 | 2.907  3.650 | 0.736  0.995 |
| **Gender**  Female=165  Male=65 | | -0.878 | 0.510 | -1.883 | 0.127 | 0.086 |
| **Role Modeling** | | | | | | |
| **YrsExp1**  [0-10] =157  [11-20] =61  [21-30] =12 | | 0.501  2.523 | 0.389  0.902 | -0.265  0.745 | 1.268  4.301 | 0.199  **0.006*** |
| **CompCE1**  [0-25] =126  [26-50] =50  [51-75] =20  [76-100] =34 | | 0.008  -1.092  -0.023 | 0.266  0.379  0.312 | -0.517  -1.840  -0.637 | 0.533  -0.345  0.591 | 0.977  **0.004***  0.940 |
| **YearGrad1**  1989-1999=14  2000-2010=63  2011-2020=153 | | 1.944  2.116 | 0.814  0.845 | 0.339  0.450 | 3.550  3.781 | **0.018***  **0.013*** |
| **Gender**  Female=165  Male=65 | | -0.489 | 0.233 | -0.950 | -0.029 | **0.037*** |
| **Team Working Skills** | | | | | | |
| **Gender**  Female=165  Male=65 | | -0.153 | 0.129 |  |  | 0.235 |
| **UniHighDeg**  OtherLeb=19  LAU=38  LIU=37  Saint Joseph University=45  Beirut Arab University=30  Lebanese University=40  OtherForeign=19 | | 0.170  -0.221  -0.078  -0.114  0.421  0.210 | 0.232  0.231  0.228  0.243  0.231  0.265 |  |  | 0.464  0.340  0.731  0.640  0.070  0.428 |
| **Whrs/dy** | | -0.063 | 0.022 |  |  | **0.004*** |
| **Pharmacist Preparedness and Response in Emergency Situations** | | | | | | |
| **Emergency Preparedness and Response** | | | | | | |
| **BS**  No=138  Yes=92 | | 1.035 | 0.973 | -0.883 | 2.953 | 0.289 |
| **CompUnderGrad1**  [0-25] =107  [26-50] =79  [51-75] =29  [76-100] =15 | | -0.074  0.027  3.505 | 0.736  1.074  1.409 | -1.525  -2.090  0.727 | 1.377  2.144  6.284 | 0.920  0.980  **0.014*** |
| **CompPostGrad1**  [0-25] =109  [26-50] =46  [51-75] =31  [76-100] =44 | | 0.728  2.310  0.654 | 0.811  1.097  1.078 | -0.870  0.146  -1.472 | 2.326  4.473  2.780 | 0.370  **0.036***  0.545 |
| **CompCE1**  [0-25] =126  [26-50] =50  [51-75] =20  [76-100] =34 | | 0.749  -1.584  0.460 | 0.767  1.212  1.210 | -0.763  -3.975  -1.927 | 2.261  0.806  2.847 | 0.330  0.193  0.704 |
| **Work2**  OtherFieldPh=29  NoOtherFieldofWrk=192  OtherField | | -1.848  -3.364 | 0.901  1.702 | -3.625  -6.719 | -0.071  -0.009 | **0.042***  **0.049*** |
| **UniPh**  Foreign University=18  LAU=46  LIU=46  Saint Joseph University=46  Beirut Arab University=39  Lebanese University=32 | | 0.841  -2.172  -0.883  -1.468  -0.626 | 1.250  1.255  1.269  1.276  1.366 | -1.623  -4.646  -3.384  -3.983  -3.319 | 3.305  0.301  1.618  1.047  2.067 | 0.502  0.085  0.487  0.251  0.647 |
| **Operation Management** | | | | | | |
| **YrsExp1**  [0-10] =157  [11-20] =61  [21-30] =12 | | -0.554  1.264 | 1.600  3.511 | -3.707  -5.664 | 2.599  8.192 | 0.729  0.719 |
| **PharmD/DPharm**  No=138  Yes=92 | | -3.850 | 1.684 | -7.172 | -0.527 | **0.023*** |
| **Master**  No=158  Yes=72 | | -3.850 | 1.502 | -3.200 | 2.727 | 0.875 |
| **Lang**  Other=6  English=146  French=78 | | -1.397  -3.732 | 3.036  4.170 | -7.389  -11.960 | 4.593  4.495 | 0.646  0.372 |
| **UniPh**  Foreign University=18  LAU=46  LIU=46  Saint Joseph University=46  Beirut Arab University=39  Lebanese University=32 | | -1.084  -3.583  0.715  -1.237  1.795 | 3.037  2.933  3.884  3.046  3.652 | -7.077  -9.371  -6.947  -7.247  -5.357 | 4.908  2.204  8.378  4.773  8.948 | 0.721  0.223  0.854  0.685  0.621 |
| **HighDeg**  BS=94  PharmD/DPharm=34  Master=52  PhD or equivalent=6  Others=4 | | 2.606  -0.742  1.205  -2.728 | 1.833  1.895  2.741  3.531 | -1.010  -4.482  -4.202  -9.696 | 6.222  2.997  6.613  4.239 | 0.157  0.696  0.660  0.441 |
| **UniHighDeg**  OtherLeb=19  LAU=38  LIU=37  Saint Joseph University=45  Beirut Arab University=30  Lebanese University=40  OtherForeign=19 | | 1.393  0.990  -0.082  -0.010  -0.354  0.466 | 2.722  2.577  2.398  2.934  2.303  2.471 | -3.797  -4.094  -4.814  -5.799  -4.898  -4.409 | 6.764  6.073  4.650  5.779  4.189  5.341 | 0.609  0.701  0.973  0.997  0.878  0.850 |
| **Work2**  OtherFieldPh=29  NoOtherFieldofWrk=192  OtherField | | -2.893  -6.555 | 1.269  2.332 | -5.397  -11.156 | -0.390  -1.953 | **0.024***  **0.005*** |
| **WLoc**  Beirut=121  Beqaa=12  Mount Lebanon=44  North Lebanon=29  South Lebanon=8  Currently not working=16 | | 0.782  2.658  0.275  0.996  3.710 | 1.902  1.089  1.331  2.343  1.732 | -2.970  0.510  -2.350  -3.627  0.291 | 4.534  4.807  2.901  5.620  7.128 | 0.681  **0.016***  0.836  0.671  **0.033*** |
| **CompUnderGrad1**  [0-25] =107  [26-50] =79  [51-75] =29  [76-100] =15 | | 0.900  1.439  2.426 | 1.087  1.485  1.994 | -1.244  -1.492  -1.508 | 3.044  4.370  6.360 | 0.409  0.334  0.225 |
| **CompPostGrad1**  [0-25] =109  [26-50] =46  [51-75] =31  [76-100] =44 | | 1.470  1.917  2.338 | 1.133  1.531  1.526 | -0.766  -1.104  -0.673 | 3.706  4.939  5.349 | 0.196  0.212  0.127 |
| **CompCE1**  [0-25] =126  [26-50] =50  [51-75] =20  [76-100] =34 | | 0.680  -1.605  -0.207 | 1.062  1.682  1.723 | -1.416  -4.924  -3.607 | 2.775  1.714  3.192 | 0.523  0.341  0.904 |
| **YearGrad1**  1989-1999=14  2000-2010=63  2011-2020=153 | | -1.372  -1.613 | 3.166  3.329 | -7.619  -8.182 | 4.875  4.955 | 0.665  0.628 |
| **CompExp1**  [0-25] =11  [26-50] =65  [51-75] =46  [76-100] =108 | | -2.476  -2.807  -2.425 | 2.105  2.195  2.068 | -6.630  -7.139  -6.504 | 1.678  1.524  1.656 | 0.241  0.202  0.242 |
| **Patient Care and Population Health interventions** | | | | | | |
| **PharmD/DPharm**  No=138  Yes=92 | | -1.048 | 0.417 | -1.871 | -0.225 | **0.013*** |
| **YearGrad1**  1989-1999=14  2000-2010=63  2011-2020=153 | | -0.877  -0.576 | 0.499  0.476 | -1.861  -1.515 | 0.107  0.362 | 0.080  0.227 |
| **HighDeg**  BS=94  PharmD/DPharm=34  Master=52  PhD or equivalent=6  Others=4 | | 0.935  -0.164  0.821  -0.145 | 0.465  0.329  0.728  0.927 | 0.018  -0.814  -0.615  -1.972 | 1.851  0.485  2.256  1.683 | 0.046  0.618  0.261  0.876 |
| **UniPh**  Foreign University=18  LAU=46  LIU=46  Saint Joseph University=46  Beirut Arab University=39  Lebanese University=32 | | -1.019  -0.977  -1.184  -1.289  -0.571 | 0.518  0.498  0.524  0.497  0.542 | -2.041  -1.959  -2.218  -2.269  -1.640 | 0.003  0.005  -0.150  -0.309  0.497 | 0.051  0.051  0.025  **0.010***  0.293 |
| **CompUnderGrad1**  [0-25] =107  [26-50] =79  [51-75] =29  [76-100] =15 | | 0.266  0.240  1.186 | 0.290  0.406  0.537 | -0.306  -0.561  0.127 | 0.838  1.042  2.245 | 0.361  0.555  **0.028*** |
| **CompPostGrad1**  [0-25] =109  [26-50] =46  [51-75] =31  [76-100] =44 | | -0.244  0.116  0.349 | 0.317  0.424  0.422 | -0.869  -0.719  -0.483 | 0.381  0.952  1.182 | 0.443  0.784  0.409 |
| **CompCE1**  [0-25] =126  [26-50] =50  [51-75] =20  [76-100] =34 | | 0.328  -0.336  1.186 | 0.294  0.464  0.464 | -0.252  -1.251  -0.903 | 0.909  0.581  0.928 | 0.266  0.471  0.978 |
| **CompExp1**  [0-25] =11  [26-50] =65  [51-75] =46  [76-100] =108 | | -0.858  -0.882  -0.702 | 0.550  0.566  0.539 | -1.942  -1.998  -1.764 | 0.225  0.234  0.360 | 0.120  0.121  0.194 |
| **Evaluation, Research, and Dissemination for Impact and Outcomes** | | | | | | |
| **UniPh**  Foreign University=18  LAU=46  LIU=46  Saint Joseph University=46  Beirut Arab University=39  Lebanese University=32 | | -1.263  -2.355  -1.441  -1.795  -1.109 | 1.021  0.988  1.076  1.010  1.117 | -3.277  -4.304  -3.562  -3.787  -3.311 | 0.750  -0.407  0.680  0.196  1.094 | 0.217  **0.018***  0.182  0.077  0.322 |
| **PharmD/DPharm**  No=138  Yes=92 | | -1.835 | 0.862 | -3.534 | -0.135 | **0.034*** |
| **HighDeg**  BS=94  PharmD/DPharm=34  Master=52  PhD or equivalent=6  Others=4 | | 1.491  -0.363  2.821  2.395 | 0.960  0.667  1.503  1.910 | -0.402  -1.678  -0.143  -1.370 | 3.384  0.952  5.784  6.161 | 0.122  0.587  0.062  0.211 |
| **CompUnderGrad1**  [0-25] =107  [26-50] =79  [51-75] =29  [76-100] =15 | | 0.822  0.714  2.417 | 0.597  0.839  1.112 | -0.355  -0.940  0.224 | 1.998  2.368  4.609 | 0.170  0.396  **0.031*** |
| **CompPostGrad1**  [0-25] =109  [26-50] =46  [51-75] =31  [76-100] =44 | | -1.552  0.099  0.894 | 0.654  0.866  0.872 | -2.841  -1.609  -0.826 | -0.263  1.808  2.614 | **0.018***  0.909  0.307 |
| **CompCE1**  [0-25] =126  [26-50] =50  [51-75] =20  [76-100] =34 | | 1.275  -0.850  -0.341 | 0.654  0.866  0.872 | 0.084  -2.739  -2.232 | 2.465  1.038  1.550 | **0.018***  0.909  0.307 |
| **CompExp1**  [0-25] =11  [26-50] =65  [51-75] =46  [76-100] =108 | | -0.566  -0.765  0.198 | 1.137  1.171  1.114 | -2.808  -3.074  -1.998 | 1.676  1.543  2.395 | 0.619  0.514  0.859 |
